# Imaging of intracranial aneurysms in animals: a systematic review of modalities

**DOI:** 10.1007/s10143-023-01953-1

**Published:** 2023-02-14

**Authors:** Anne F. Cayron, Sandrine Morel, Eric Allémann, Philippe Bijlenga, Brenda R. Kwak

**Affiliations:** 1 Department of Pathology and Immunology, Faculty of Medicine, University of Geneva, Rue Michel-Servet 1, CH-1211 Geneva, Switzerland; 2 Geneva Center for Inflammation Research, Faculty of Medicine, University of Geneva, Geneva, Switzerland; 3 School of Pharmaceutical Sciences, University of Geneva, Geneva, Switzerland; 4 Institute of Pharmaceutical Sciences of Western Switzerland, University of Geneva, Geneva, Switzerland; 5 Department of Clinical Neurosciences - Division of Neurosurgery, Geneva University Hospitals and Faculty of Medicine, University of Geneva, Geneva, Switzerland

**Keywords:** Intracranial aneurysm, Animal models, Diagnostic imaging, Diagnostic technique

## Abstract

Intracranial aneurysm (IA) animal models are paramount to study IA pathophysiology and to test new endovascular treatments. A number of in vivo imaging modalities are available to characterize IAs at different stages of development in these animal models. This review describes existing in vivo imaging techniques used so far to visualize IAs in animal models. We systematically searched for studies containing in vivo imaging of induced IAs in animal models in PubMed and SPIE Digital library databases between 1 January 1945 and 13 July 2022. A total of 170 studies were retrieved and reviewed in detail, and information on the IA animal model, the objective of the study, and the imaging modality used was collected. A variety of methods to surgically construct or endogenously induce IAs in animals were identified, and 88% of the reviewed studies used surgical methods. The large majority of IA imaging in animals was performed for 4 reasons: basic research for IA models, testing of new IA treatment modalities, research on IA in vivo imaging of IAs, and research on IA pathophysiology. Six different imaging techniques were identified: conventional catheter angiography, computed tomography angiography, magnetic resonance angiography, hemodynamic imaging, optical coherence tomography, and fluorescence imaging. This review presents and discusses the advantages and disadvantages of all in vivo IA imaging techniques used in animal models to help future IA studies finding the most appropriate IA imaging modality and animal model to answer their research question.

## Introduction

Intracranial aneurysm (IA) is an arterial disease resulting in abnormal enlargement of the vessel lumen. IAs generally form at bifurcations of intracranial arteries in the circle of Willis, an arterial network that supplies blood to the brain [1, 2]. IAs affect 2 to 5% of the population and are mostly asymptomatic [3, 4]. However, unstable IAs can rupture causing subarachnoid hemorrhage (SAH) that is fatal in 25–50% of cases [5]. Moreover, 35% of the patients that survive SAH suffer from long-term sequelae such as physical or cognitive disabilities impairing their quality of life [6]. To reduce this risk, the decision to secure the aneurysm by surgical clipping, endovascular coiling, and/or flow diversion could be taken [5]. In vivo imaging techniques are of paramount importance in the management of IAs. Indeed, high-resolution imaging is needed to assess precisely size and morphology of the IA (presence of blebs, lobules, rough aspect), which is necessary to evaluate the risk of rupture of an IA at its discovery and during follow-up imaging [7, 8]. IAs of large diameter or displaying irregular vessel walls have been linked to an increased risk of rupture. These features are commonly used in the different IA risk scoring systems, even if the biological processes leading to IA rupture is still unknown [9–11]. Longitudinal studies to understand the pathophysiology underlying the relation between these morphological features and the increased risk of rupture require research on IA animal models. Such models also allow for the testing of new endovascular treatments.

In this context, various IA animal models have been established. The first induced IAs in animals were surgically constructed to mimic IAs in human cerebral arteries [12]. Already in 1954, German and Black [13] induced IAs by grafting a vein-pouch into the common carotid artery (CCA) of dogs. Thereafter, different venous pouch models allowing for the formation of aneurysms with adaptable sizes, at different locations, and with various shapes have been used in several species [12]. Altes et al. [14] developed a rabbit model of IAs located at the origin of the right CCA using elastase incubation and ligation of the right CCA. Later, endogenous IA animal models mimicking the human disease helped to better understand IA pathogenesis and development [15]. Different risk factors for the formation of IAs have been identified and can be used in animals to endogenously induce IAs. Hemodynamic stress in combination with other vascular risk factors, such as hypertension, is known to be involved in the initiation of IA formation [16]. One component of hemodynamic stress is wall shear stress (WSS), which is the drag force exerted by blood flow onto the endothelium, the innermost layer of the vessel wall. High and low WSS seem both involved in the progression or growth of IAs [2]. Connective tissue disorders like the Ehlers-Danlos syndrome and the Marfan syndrome also put patients more at risk to develop IAs [1]. Thus, chemical compounds that weaken connective tissue extracellular matrix components such as β-aminopropionitrile (BAPN) and elastase are used in animals to favor IA formation [15]. These endogenous IA animal models mimic arterial wall modifications that characterize the human disease, such as loss of the internal elastic lamina, loss of endothelial and smooth muscle cells, as well as inflammatory cell infiltration [16].

To study the size and morphology at different stages of IA development in these surgical and endogenous animal models, in vivo IA imaging is necessary. Like in human, angiography is used in animals to image the IA and, more particularly, to assess the patency of induced IAs over time or after endovascular treatment [17]. The same imaging modalities have been used in human and animal models; however, IA imaging in animal models is challenging, as many induced IAs are smaller than human IAs. [15]. Digital subtraction angiography (DSA) remains the gold standard imaging technique, but progress in imaging technologies increased image resolution, and many different imaging modalities are currently available to image IAs in vivo in animals [18]. We performed a systematic review to list all imaging techniques used in IA animal models to help researchers finding the most appropriate IA imaging modality to answer their research question. In this review, we first describe briefly all existing in vivo imaging techniques used for IA visualization in animal studies. Then, we critically compare the different imaging modalities and discuss their advantages and disadvantages.

## Methods

### Search strategy

**Fig. 1:**
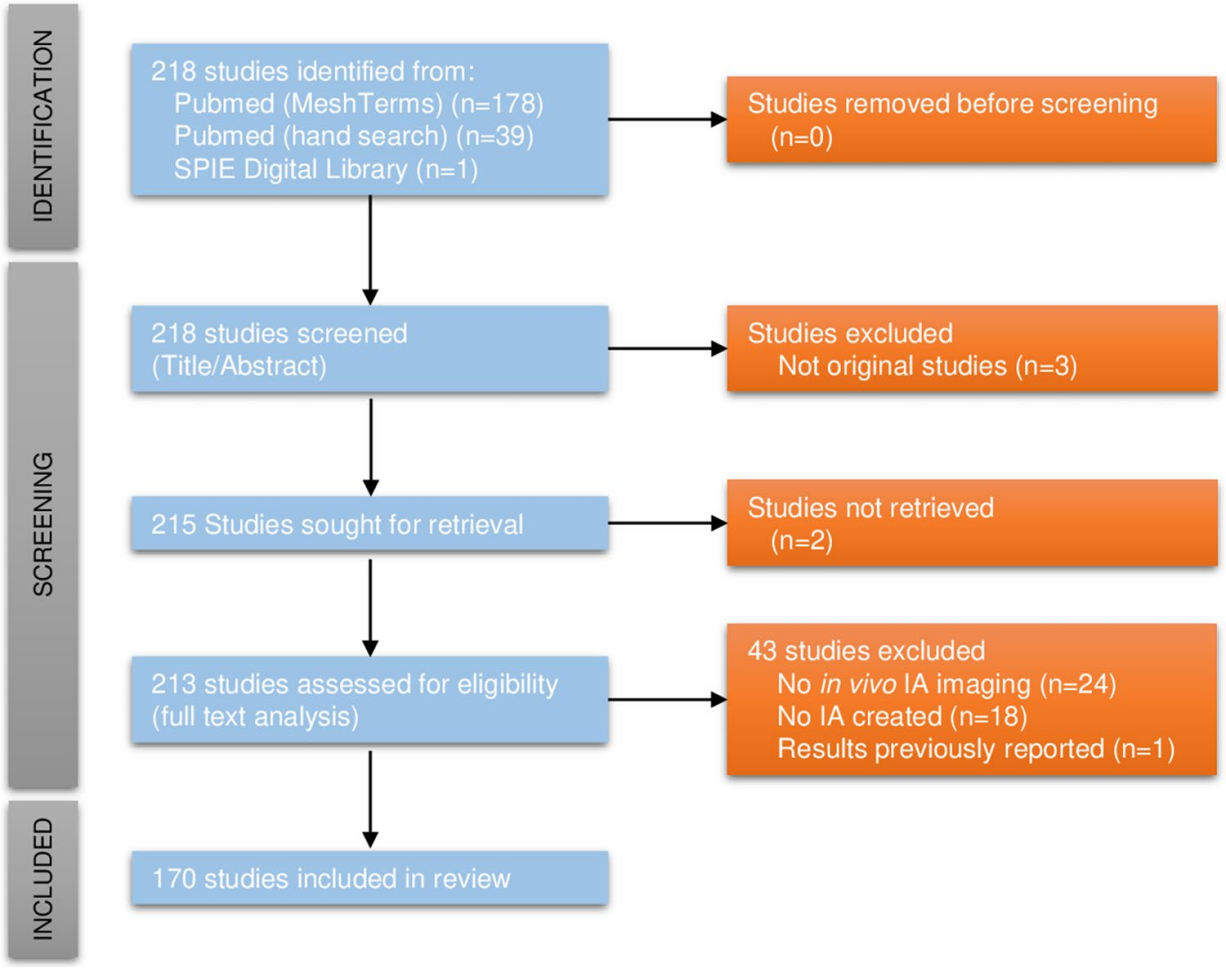
Preferred Reporting Items for Systematic Reviews and Meta-Analyses (PRISMA) flow diagram. Two hundred eighteen studies were identified on PubMed and SPIE Digital Library, and no duplicate were found. Three studies were excluded after title/abstract screening as they were not original studies. Full texts of 213 out of 215 studies were available and retrieved. Forty-three studies were excluded after full text analysis according to the exclusion criteria, yielding 170 studies included in this review

We systematically searched for studies in PubMed between 1 January 1945 and 13 July 2022 containing in vivo imaging of IA in animal models. We used the combination of the following Medical Subject Headings (MeSH) terms: “intracranial aneurysm” AND “animal models” AND (“diagnostic imaging” OR “diagnostic technique, cardiovascular”) and excluded reviews. A hand search in the PubMed database and in the SPIE Digital library was also performed to find studies not found with the MeSH terms cited above. Then, potentially eligible studies were screened and included or excluded from this review following the Preferred Reporting Items for Systematic Reviews and Meta-Analyses (PRISMA) guidelines [19] (Fig. 1). Included studies were carefully examined, and information on the objective(s) of the study, the IA animal model(s), and the imaging modality(ies) used was collected (Table 1).

**Table 1:** Included studies in the review for in vivo imaging in IA animal models

Articles, in publication date order		Objective(s) of the study					IA animal models			Imaging modality(ies)					
First author	Year	Basic research for IA models	Testing of new IA treatment modalities	Research on in vivo imaging of IAs	Research on IA pathophysiology	Other	Endogenous or surgical	Animal species	IA type	Conventional catheter angiography	Computed tomography angiography	Magnetic resonance angiography	Hemodynamic imaging	Optical coherence tomography	Fluorescence imaging
German W.J. [13]	1954	X					Surgical	Dogs	Saccular	X					
Black S.P. [20]	1960	X					Surgical	Dogs	Saccular	X					
Roy V.C. [21]	1970					X	Surgical	Monkeys	Saccular	X					
Cares H.L. [22]	1973		X				Surgical	Dogs	Saccular	X					
Maira G. [23]	1979		X				Surgical	Rabbits	Saccular	X					
Nagata I. [24]	1979			X			Endogenous	Rats	Saccular	X					
Sandor T. [25]	1980			X			Surgical	Rabbits	Saccular	X					
O'Reilly G.V. [26]	1981	X					Surgical	Rabbits	Saccular	X					
Hashimoto N. [27]	1984				X		Endogenous	Rats	Saccular	X					
Hashimoto N. [28]	1987	X					Endogenous	Monkeys	Saccular	X					
Forrest M.D. [29]	1989	X					Surgical	Rabbits	Saccular	X					
Nakatani H. [30]	1993			X			Endogenous	Rats	Saccular						X
Hashimoto T. [31]	1993				X		Surgical	Rabbits	Saccular				X		
Graves V.B. [32]	1993	X					Surgical	Dogs	Saccular	X					
Massoud T.F. [33]	1994	X					Surgical	Swines	Saccular	X					
Geremia G. [34]	1994		X				Surgical	Dogs	Saccular	X					
Guglielmi G. [35]	1994	X					Surgical	Swines	Saccular	X					
Wakhloo A.K. [36]	1994		X				Surgical	Dogs	Saccular	X					
Cawley C.M. [37]	1996	X					Surgical	Rabbits	Saccular	X					
Kirse D.J. [38]	1996	X					Surgical	Rats	Saccular	X		X			
Spetzger U. [39]	1996	X					Surgical	Rabbits	Saccular	X					
Bavinzski G. [40]	1998	X					Surgical	Rabbits	Saccular	X					
Fukui K. [41]	1998	X					Surgical	Rats	Fusiform	X			X		
Kallmes D.F. [42]	1999	X					Surgical	Dogs	Saccular	X			X		
Kallmes D.F. [43]	1999		X				Surgical	Rabbits	Saccular	X					
Ujiie H. [44]	1999				X		Surgical	Rabbits	Saccular				X		
Cloft H.J. [45]	1999	X					Surgical	Rabbits	Saccular	X					
Murayama Y. [46]	1999		X				Surgical	Swines	Saccular	X					
Altes T.A. [14]	2000	X					Surgical	Rabbits	Saccular	X					
Murayama Y. [47]	2001		X				Surgical	Swines	Saccular	X					
Short J.G. [48]	2001	X					Surgical	Rabbits	Saccular	X					
de Gast A.N. [49]	2001		X				Surgical	Rabbits	Saccular	X					
Fujiwara N.H. [50]	2001	X					Surgical	Rabbits	Saccular	X					
Raymond J. [51]	2002		X				Surgical	Dogs	Saccular	X					
Kallmes D.F. [52]	2002	X					Surgical	Rabbits	Saccular	X					
Fujiwara N.H. [53]	2002		X				Surgical	Rabbits	Saccular	X					
Raymond J. [54]	2002		X				Surgical	Dogs	Saccular	X					
Kallmes D.F. [55]	2002		X				Surgical	Rabbits	Saccular	X					
Krings T. [56]	2002			X			Surgical	Rabbits	Saccular	X		X			
Kallmes D.F [57]	2003		X				Surgical	Rabbits	Saccular	X					
Raymond J. [58]	2003		X				Surgical	Swines and dogs	Saccular	X					
Krings T. [59]	2003	X					Surgical	Rabbits	Saccular	X		X			
Murayama Y. [60]	2003		X				Surgical	Swines	Saccular	X					
Möller-Hartmann W. [61]	2003	X					Surgical	Rabbits	Saccular	X					
Thiex R. [62]	2004	X					Surgical	Rabbits	Saccular	X					
Doerfler A. [18]	2004			X			Surgical	Rabbits	Saccular	X	X	X			
Raymond J. [63]	2004	X					Surgical	Dogs	Saccular	X					
Yoshino Y [64]	2004		X				Surgical	Dogs	Saccular	X					
Hoh B.L. [65]	2004	X					Surgical	Rabbits	Saccular	X					
Seong J. [66]	2005					X	Surgical	Rabbits	Saccular	X					
Thorell W.E. [67]	2005			X			Surgical	Dogs	Saccular	X				X	
Shin Y.S. [68]	2005	X					Surgical	Dogs	Saccular	X					
Ding Y.H. [69]	2005	X					Surgical	Rabbits	Saccular	X					
Raymond J. [70]	2005				X		Surgical	Dogs	Saccular	X					
Grunwald I.Q. [71]	2005		X				Surgical	Rabbits	Saccular	X					
Boulos A.S. [72]	2005	X					Surgical	Dogs	Saccular	X					
Ding Y.H. [73]	2006	X					Surgical	Rabbits	Saccular	X					
Sadasivan C. [74]	2006		X				Surgical	Rabbits	Saccular	X					
Onizuka M. [75]	2006	X					Surgical	Rabbits	Saccular	X					
Ding Y.H. [76]	2006	X					Surgical	Rabbits	Saccular	X					
Acar F. [77]	2006		X				Surgical	Rabbits	Saccular	X					
Ding Y.H. [78]	2006			X			Surgical	Rabbits	Saccular	X					
Dudeck O. [79]	2006		X				Surgical	Swines	Saccular	X	X	X			
Dudeck O. [80]	2006		X				Surgical	Swines	Saccular	X	X				
Frösen J. [81]	2006				X		Surgical	Rats	Saccular			X			
Dai D. [82]	2006		X				Surgical	Rabbits	Saccular	X					
Dai D. [83]	2007		X				Surgical	Rabbits	Saccular	X					
Song J.K. [84]	2007		X				Surgical	Dogs	Saccular	X					
Ding Y.H. [85]	2007	X					Surgical	Rabbits	Saccular	X					
Kallmes D.F. [86]	2007		X				Surgical	Rabbits	Saccular	X					
Ahlhelm F. [87]	2007	X					Surgical	Rabbits	Saccular	X					
Kadirvel R. [88]	2007				X		Surgical	Rabbits	Saccular	X			X		
Turk A.S. [89]	2008		X				Surgical	Dogs	Saccular	X					
Tsumoto T. [90]	2008	X					Surgical	Dogs	Saccular	X					
Gao L. [91]	2008				X		Endogenous	Rabbits	Saccular	X			X		
Struffert T. [92]	2008		X				Surgical	Rabbits	Saccular	X					
Arends J. [93]	2008	X					Surgical	Swines	Saccular	X					
Berenstein A. [94]	2009		X				Surgical	Dogs	Saccular	X					
Sadasivan C. [95]	2009		X				Surgical	Rabbits	Saccular	X					
Marjamaa J. [96]	2009		X				Surgical	Rats	Saccular			X			
Tsumoto T. [97]	2009		X				Surgical	Dogs	Saccular	X					
Wang Z. [98]	2009				X		Endogenous	Dogs	Saccular	X			X		
Killer M. [99]	2009		X				Surgical	Rabbits	Saccular	X					
Takao H. [100]	2009		X				Surgical	Swines	Saccular	X					
DeLeo M.J. 3^rd^ [101]	2009			X			Surgical	Rabbits	Saccular	X		X			
Struffert T. [102]	2010			X			Surgical	Rabbits	Saccular	X	X				
Reinges M.H. [103]	2010		X				Surgical	Rabbits	Saccular	X					
Killer M. [104]	2010		X				Surgical	Rabbits	Saccular	X					
Sherif C. [105]	2011	X					Surgical	Rabbits	Saccular	X					
Zeng Z. [106]	2011	X					Surgical	Rabbits	Saccular	X			X		
Marbacher S. [107]	2011	X					Surgical	Rabbits	Saccular	X		X			
Gupta R. [108]	2011			X			Surgical	Rabbits	Saccular		X				
Jiang J. [109]	2011			X			Surgical	Dogs	Saccular	X		X	X		
Kolega J. [110]	2011				X		Endogenous	Rabbits	Saccular	X			X		
Cai J. [111]	2012	X					Endogenous	Rats	Saccular	X					
Ysuda R. [112]	2012	X					Surgical	Dogs	Saccular	X					
Spilberg G. [113]	2012			X			Surgical	Dogs	Saccular	X		X			
Darsaut T.E. [114]	2012		X				Surgical	Dogs	Saccular	X					
Darsaut T.E. [115]	2012		X				Surgical	Dogs	Saccular	X					
Marbacher S. [116]	2012	X					Surgical	Rabbits	Saccular			X			
Mühlenbruch G. [117]	2013	X					Surgical	Swines	Saccular	X					
Struffert T. [118]	2013		X				Surgical	Rabbits	Saccular	X			X		
Raymond J. [119]	2013				X		Surgical	Swines	Saccular	X					
Wang J.B. [120]	2013		X				Surgical	Dogs	Saccular	X					
Turk A. [121]	2013		X				Surgical	Dogs	Saccular	X					
Brennecka C.R. [122]	2013		X				Surgical	Dogs	Saccular	X					
Huang Q. [123]	2013		X				Surgical	Rabbits	Saccular	X					
Wang Y. [124]	2013	X					Surgical	Rabbits	Saccular	X					
Kühn A.L. [125]	2014		X				Surgical	Rabbits	Saccular	X					
Simgen A. [126]	2014		X				Surgical	Rabbits	Saccular	X					
Mitome-Mishima Y. [127]	2014		X				Surgical	Swines	Saccular	X					
Liu Y. [128]	2014				X		Surgical	Rabbits	Saccular and fusiform		X				
Donzelli R. [129]	2014		X				Surgical	Rabbits	Saccular	X					
Erhardt S. [130]	2014					X	Surgical	Rabbits	Saccular			X			
Cebral J.R. [131]	2014		X				Surgical	Rabbits	Saccular	X			X		
Wang J. [132]	2014				X		Endogenous	Dogs	Saccular	X			X		
Zhu Y.Q. [133]	2014				X		Endogenous	Dogs	Saccular	X			X		
Cebral J.R. [134]	2014		X				Surgical	Rabbits	Saccular	X			X		
Darsaut T.E. [135]	2014		X				Surgical	Dogs	Saccular	X					
Marbacher S. [136]	2014				X		Surgical	Rats	Saccular			X		X	
Marbacher S. [137]	2014				X		Surgical	Rats	Saccular			X			
Gounis M.J. [138]	2015			X			Surgical	Rabbits	Saccular	X		X			
Sherif C. [139]	2015			X			Surgical	Rabbits	Saccular			X			
Chavan R. [140]	2015		X				Surgical	Rabbits	Saccular	X					
Krähenbühl A.K. [141]	2015	X					Surgical	Rabbits	Saccular	X					
Ott S. [142]	2015			X			Surgical	Rabbits	Saccular	X	X				
Makino H. [143]	2015			X			Endogenous	Mice	Saccular			X			
Jiang Y.Z. [144]	2015	X					Surgical	Dogs	Saccular	X			X		
Puffer C. [145]	2015		X				Surgical	Rabbits	Saccular	X					
Tutino V.M. [146]	2016	X					Endogenous	Rats and rabbits	Saccular	X		X	X		
Yuki I. [147]	2016			X			Surgical	Swines	Saccular	X	X				
Rouchaud A. [148]	2016		X				Surgical	Rabbits	Saccular	X					
Ding Y. [149]	2016		X				Surgical	Rabbits	Saccular	X					
Miura Y. [150]	2016		X				Surgical	Rabbits	Saccular	X					
Ding Y.H. [151]	2016		X				Surgical	Rabbits	Saccular	X					
Brinjikji W. [152]	2017		X				Surgical	Rabbits	Saccular	X					
Fahed R. [153]	2017		X				Surgical	Dogs	Saccular	X					
Miyamoto T. [154]	2017				X		Endogenous	Rats	Saccular				X		
Adibi A. [155]	2017		X				Surgical	Rabbits	Saccular	X					
Tutino V.M. [156]	2018			X			Endogenous	Mice	Saccular			X	X		
Greim-Kuczewski K. [157]	2018	X					Surgical	Rabbits	Saccular	X					
Marotta T.R. [158]	2018		X				Surgical	Rabbits	Saccular	X					
Li Z.F. [159]	2018				X		Surgical	Rabbits	Saccular	X					
Fahed R. [160]	2018		X				Surgical	Dogs	Saccular	X					
King R.M. [161]	2018		X				Surgical	Rabbits	Saccular	X				X	
Caroff J. [162]	2018		X				Surgical	Rabbits	Saccular	X				X	
Marosfoi M. [163]	2018		X				Surgical	Rabbits	Saccular	X				X	
Miyata H. [164]	2019			X			Endogenous	Rats	Saccular						X
Liu Y. [165]	2019			X			Surgical	Rabbits	Saccular	X				X	
Simgen A. [166]	2019			X			Surgical	Rabbits	Saccular	X					
Strange F. [167]	2019			X			Surgical	Rats and rabbits	Saccular						X
Ikedo T. [168]	2019				X		Endogenous	Rats	Saccular			X			
Wang G.X. [169]	2019			X			Surgical	Rabbits	Saccular		X	X			
Grüter B.E. [170]	2019			X			Surgical	Rats	Saccular						X
Nishi H. [171]	2019		X				Surgical	Rabbits	Saccular	X				X	
Iseki S. [172]	2019		X				Surgical	Swines	Saccular	X					
King R.M. [173]	2019			X			Surgical	Dogs	Saccular	X	X			X	
Rajabzadeh-Oghaz H. [174]	2019			X			Endogenous	Mice	Saccular			X			
Wanderer S. [175]	2020	X					Surgical	Rabbits	Saccular			X			X
Lyu Y. [176]	2020	X					Surgical	Rabbits	Fusiform	X			X		
Fries F. [177]	2020			X			Surgical	Rabbits	Saccular	X				X	
Sun X. [178]	2020				X		Endogenous	Rabbits	Saccular				X		
Koseki H. [179]	2020				X		Endogenous	Rats	Saccular						X
Ho J.P. [180]	2021				X		Surgical	Rabbits	Saccular			X			
Shimizu K. [181]	2021	X					Endogenous	Rats	Saccular			X	X		
Vardar Z. [182]	2021		X				Surgical	Rabbits	Saccular	X				X	
Zhang Y. [183]	2021			X			Surgical	Rabbits	Saccular	X		X			
Fries F. [184]	2022		X				Surgical	Rabbits	Saccular	X					
Hufnagl C. [185]	2022		X				Surgical	Rabbits	Saccular	X					
Wanderer S. [186]	2022			X			Surgical	Rabbits	Saccular			X			X

The 170 studies are presented chronologically, and information on the objective(s) of the study, the IA animal models and the imaging modality(ies) employed are reported

### Eligibility criteria

Exclusion criteria were defined by the objective of this study to review only articles containing in vivo IA imaging in animal models. Articles that did comprise the following criteria were not included in this review: (1) no in vivo imaging, (2) no IA created, and (3) results previously reported. Studies in which IA imaging was performed after sacrifice of the animal were also not included as this review focuses on in vivo imaging modalities that can be used in living animals. However, animal models using extracranial aneurysms as an IA animal model were included in this review as they mimic human IAs in terms of aneurysm size and vessel diameter.

## Results

A total of 178 potentially eligible studies were found in PubMed using MeSH terms and 39 after the hand search (Fig. 1). Moreover, 1 additional potentially eligible study was found on SPIE Digital library. After the screening of the title and the abstract of these 218 studies, 3 articles were excluded as they were not original studies. Full texts from 2 studies were not available and had to be excluded for this reason. Following the exclusion criteria defined above, 43 articles were withdrawn from the review, yielding 170 studies included in this review (Table 1).

### IA animal models

IA animal models are required to test new endovascular devices and to better understand IA pathophysiology. However, spontaneous endogenous cerebral aneurysms are extremely rare in animals [187]. Consequently, many techniques to induce IAs in various animal species were successfully established. Large animal models like swines and dogs are well-characterized IA animal models with an easy access for diagnostic and IA treatment; however, they are expensive models [188]. Rabbit IA models are also well characterized and commonly used as their carotid artery size is comparable to human cerebral arteries [189]. Unfortunately, rabbits have a relatively high perioperative morbidity and mortality [188]. Finally, the use of small rodents, like mice and rats, allow for lower study costs, but their arteries are much smaller than human cerebral arteries, making surgery and imaging more difficult and not adapted to human endovascular techniques [188].

**Fig. 2:**
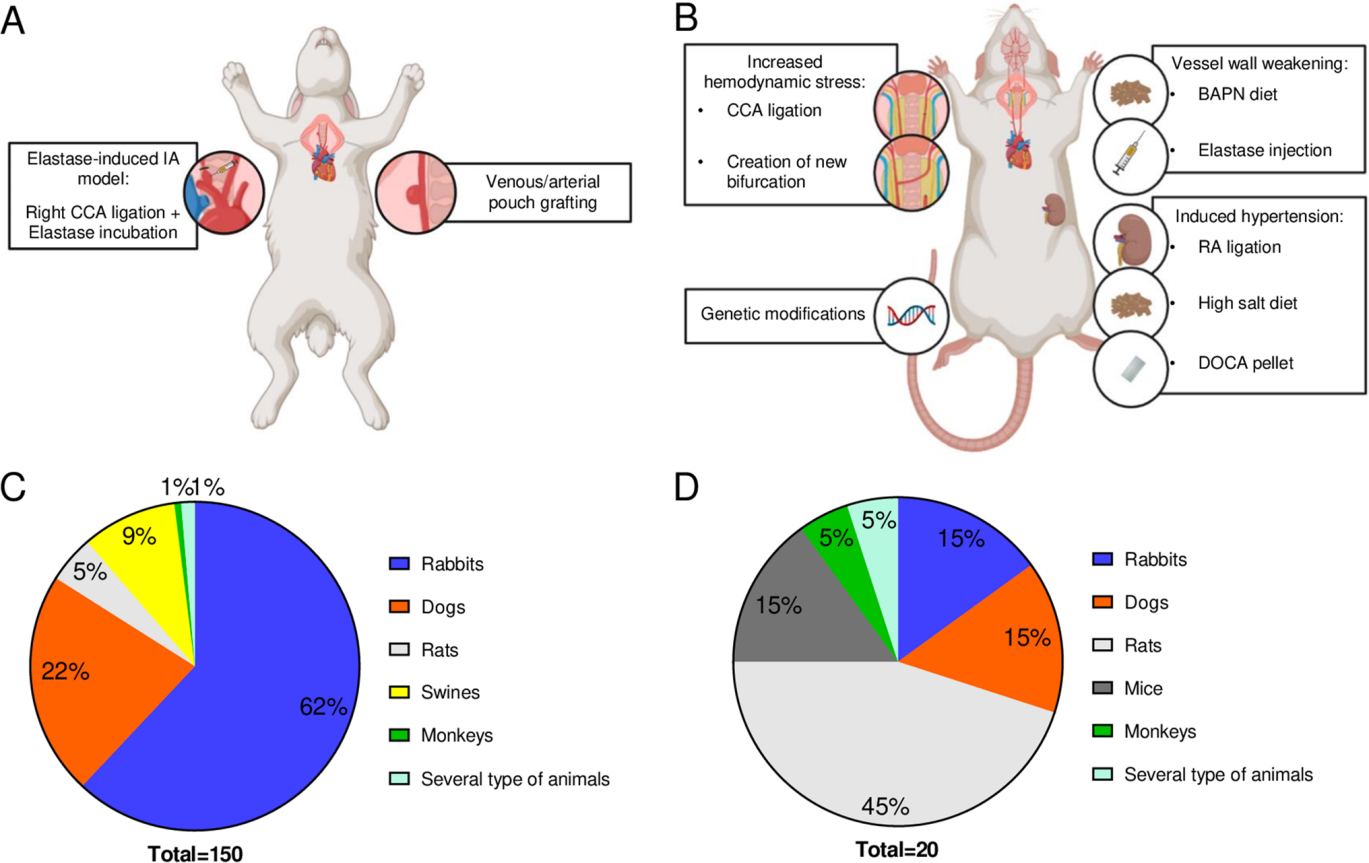
IA animal models can be divided in surgical and endogenous models. **A** Surgical IAs can be constructed using venous or arterial pouch grafting or elastase incubation in combination with right CCA ligation. **B** Endogenous IA models comprise models using IA risk factors such as increased hemodynamic stress, vessel wall weakening, induced hypertension, or genetic modifications. **C** and **D** The distribution of animal species is different between surgical (**C**) and endogenous (**D**) IA animal models in the 170 reviewed studies. Rabbit is the most frequently used animal for surgically constructed IAs, whereas rats are more frequently used in endogenous IA models

Different techniques to surgically construct or endogenously induce IAs in animal models are available. In the first category, IAs are surgically constructed using venous or arterial pouch grafting (Fig. 2A, right side) or using artery ligation in combination with vessel wall weakening using elastase like in the frequently used rabbit elastase model [188] (Fig. 2A, left side). In the second category of endogenously induced IA animal models, animals are exposed to known IA risk factors such as hemodynamic stress or connective tissue weakening [15] (Fig. 2B). Hemodynamic stress can be increased by unilateral or bilateral CCA ligation or by the creation of a new bifurcation between 2 arteries. Therefore, hemodynamic stress can be increased in specific extracranial arteries or in intracranial arteries of the circle of Willis. Hemodynamic stress can also be increased by inducing hypertension by ligation of one renal artery (RA), with or without high salt diet or deoxycorticosterone acetate (DOCA) pellet implantation. In addition, the vessel wall can be weakened by elastase injection or inclusion of BAPN in the food of the animals. Furthermore, genetic modifications have been occasionally used in combination with other manipulations to induce IAs [15]. The vast majority of the studies used saccular IA models, and only 2% of the studies used an animal model of fusiform IAs (Table 1).

Eighty-eight percent of the reviewed studies used surgical methods (Table 1). Indeed, in most of the studies, IAs are surgically induced in large animals like rabbits or dogs that are relatively simple to image (Fig. 2C). In contrast, only 12% of the reviewed articles used methods in which IAs are endogenously formed. Actually, endogenous IA models mainly use small animals like rats (Fig. 2D) making IA imaging more difficult due to the very small size of the induced lesions [8].

### Purpose of in vivo imaging of IAs in animal models

**Fig. 3:**
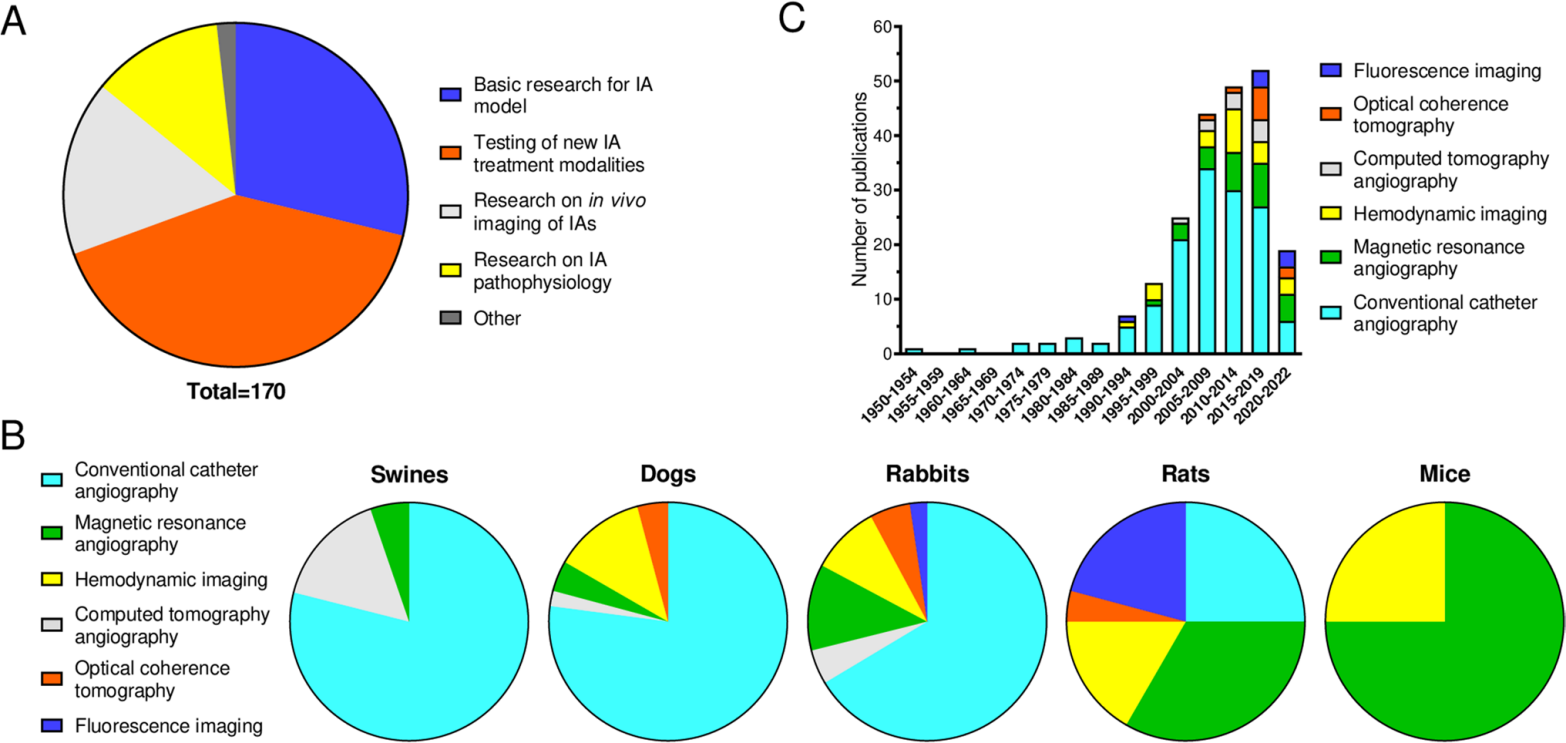
Purpose, distribution among species, and evolution of the different in vivo IA imaging modalities in animal studies. **A** Reasons for IA in vivo imaging in animals. **B** Distribution of the use of imaging modalities in swines, dogs, rabbits, rats, and mice. The two studies with monkeys used conventional catheter angiography to image IAs (not shown). **C** Number of published articles among the years using in vivo imaging in IA animal models

The large majority of IA imaging in animals was performed in the reviewed studies for 4 reasons: basic research for IA models, testing of new IA treatment modalities, research on in vivo imaging of IAs, and research on IA pathophysiology (Table 1 and Fig. 3A). The objectives of 3 studies was classified as “other” as they could not be categorized in the above classes. The vast majority of the studies included in this review focused on testing new IA treatment options (Fig. 3A). Indeed, imaging is essential during coiling or flow diverter implantation and later to check the effectiveness of the treatment over time. IA imaging is also instrumental in research on IA animal models as it allows for the visualization and characterization of IA morphology and IA patency surveillance over time. Moreover, in vivo imaging of IAs also helps to understand IA pathophysiology better as it allows to follow IA size and morphology from IA initiation to rupture, for instance. Thus, in vivo imaging of IAs is crucial in animal models. Actually, 17% of reviewed articles explicitly aimed at research on IA imaging itself.

### Description of the IA in vivo imaging techniques

The different imaging modalities found in the reviewed articles were classified in 6 different imaging techniques: conventional catheter angiography, computed tomography angiography (CTA), magnetic resonance angiography (MRA), hemodynamic imaging, optical coherence tomography (OCT), and fluorescence imaging. Pros and cons of these imaging modalities have been highlighted (Table 2). These advantages and disadvantages are likely responsible for the specific distribution of the imaging modalities among the different animal species, with conventional catheter angiography being most frequently used in larger animals, and a preference for MRA was found in smaller animals (Fig. 3B).

- **Conventional catheter angiography**

**Table 2:**
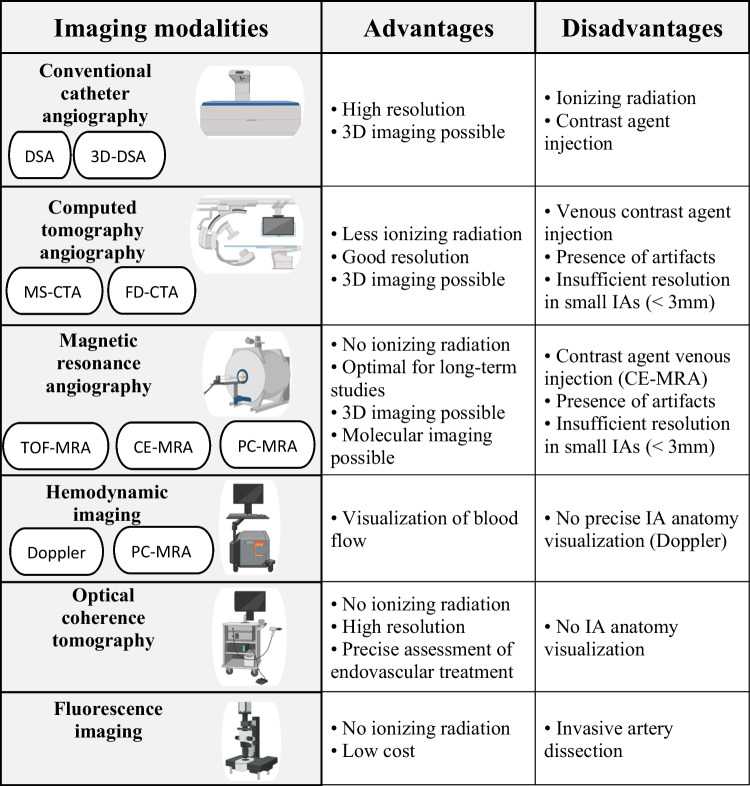
Advantages and disadvantages of the different in vivo imaging modalities

Catheter angiography is the conventional angiography method that uses X-ray and injection of a nonionic iodinated contrast agent, usually through an arterial catheter, to image blood vessels [190]. In 1954, German and Black [13] were the first to image a constructed IA in the CCA of dogs, using 2D catheter angiography. Until 1993, catheter angiography was the only available modality to image IAs in animal models (Fig. 3C). Imaging improvements continued [25] and led Wakhloo et al. [36] to use DSA to image IAs in CCA of dogs in 1994. DSA is a 2D catheter angiography that uses subtraction of precontrast images to obtain an image of the vasculature only [191]. From then on, DSA became the gold standard to detect IAs in human [192] and in animal models [18]. Actually, 84% of the reviewed studies used conventional catheter angiography (Table 1). Indeed, as mentioned before, the most frequent purpose to use in vivo imaging of IAs in animal studies is to test new endovascular treatment modalities, and 97% of these studies use catheter angiography. Conventional catheter angiography allows the correct assessment of IA occlusion following IA endovascular treatment. DSA in animal models is mainly performed through the femoral artery. However, it can also be performed through the central artery of the ear in rabbits [78] or through veins (e.g., ear vein) [18, 48, 50, 65, 71, 73, 91]. Intra-venous DSA (i.v. DSA) is less invasive and allows for repeated imaging compared to intra-arterial DSA (i.a. DSA), which requires exposure, cutdown, and ligation of arteries making serial imaging sessions more difficult and risky [50, 71]. Doerfler et al. [18] showed that i.v. DSA is as precise as i.a. DSA to efficiently predict IA size and geometry in the rabbit elastase IA model. However, other studies revealed that i.v. DSA underestimates the IA dimension [112] or is insufficient to assess aneurysm occlusion after flow diverter treatment [166] compared to i.a. DSA. The authors postulated that this difference results from a decreased contrast agent aneurysm filling due to contrast agent dilution in the bloodstream and/or decreased velocity during i.v. DSA. Moreover, it should be noted that, despite its decreased invasiveness, i.v. DSA did not replace i.a. DSA in clinic because of the decreased contrast density and the vessel overlapping visualization due to the simultaneous imaging of veins and arteries [193].

3D rotational DSA (3D-DSA) images cerebral vessels from all viewpoints, thereby increasing precision for 3D geometry assessment of arteries [194]. This allows for visualization of vascular modeling to create precise vascular devices [66, 133]. It is assumed that 3D-DSA offers the greatest resolution compared to other imaging techniques [112]. However, it has been shown in human studies that DSA displays a better resolution of small vessels, which allows a greater sensitivity in small IAs than with 3D-DSA [192].

As it provides an very high resolution allowing the visualization of small cerebral vessels (< 1 mm), e.g., the anterior choroidal artery [195], DSA continues to be the gold standard [7, 18]. However, DSA remains an invasive method using radiation and injection of contrast agent through a catheter and is associated with a complication rate of 0.04–0.30% in humans [194]. Therefore, less invasive imaging methods represent a safe alternative in human and animal models [18].

- **Computed tomography angiography**

CTA uses X-ray and contrast agent injection through a venous catheter to image the vasculature and allows for a 60% decrease of ionizing radiation making CTA less invasive than conventional catheter angiography [7]. This imaging modality uses the rotation of a CT scanner in combination with a motile patient table that allows a continuous 2D or 3D image acquisition at a higher speed than conventional catheter angiography [196, 197]. The first use of CTA in an IA animal model was described in 2004, and only 10 studies included in this review used CTA (Table 1 and Fig. 3C). Human studies revealed that CTA has an insufficient resolution in small IAs (diameter < 3 mm) [198]. However, several studies showed that CTA is as efficient as DSA to detect IAs in animal models [18, 79, 80, 102, 142].

Different types of detectors can be used with CT: multi-slice detectors (MS-CTA) and flat-detectors (FD-CTA), which have been introduced later and use a smaller detector element size. Struffert et al. [102] showed that CT with both detectors allowed for measurement of similar IA dimensions in the rabbit elastase model, but images seem to be better delineated using FD-CTA due to a higher spatial resolution.

A major limitation of CTA in human is the presence of artifacts when clips, stent, or coils are used [192]. Yet, Dudeck et al. [79] did not observe such effects in CCA aneurysms constructed in swine and coiled with a liquid embolic agent. Moreover, Ott et al. [142] observed limited coil artifacts with FD-CTA in comparison with MS-CTA. In addition, metal reduction software used on high-resolution CT scans considerably decreases stent artifacts [147].

- **Magnetic resonance angiography**

Magnetic resonance imaging allows for less invasive 2D and 3D angiography using powerful magnetic fields without ionizing radiation nor iodine-based contrast agent injection [199]. This less invasive imaging modality makes MRA an optimal instrument for serial imaging in long-term studies. Indeed, safe serial imaging in long-term studies using MRA was performed in dogs [113], mouse [143, 156, 174], rats [96, 136, 137, 181], and rabbit elastase IA models [18, 169].

Several studies in human suggest that MRA could be considered equivalent to DSA to detect IAs [7]. However, it has also been shown that the resolution was insufficient to detect small IAs (< 3 mm) [7]. Sixteen percent of the animal studies included in this review used MRA (Table 1), and this imaging modality was more often used in the last 10–15 years (Fig. 3C). In 1996, Kirse et al. [38] were the first to image IAs in a surgical rat model using MRA. In this study, however, DSA showed a better resolution than MRA. Different MRA methods are available: time of flight MRA (TOF-MRA), phase contrast MRA (PC-MRA), or contrast-enhanced MRA after venous contrast agent injection (CE-MRA) [200]. Krings et al. [56] imaged IAs in 5 rabbits using the elastase model with the 3 afore-mentioned MRA methods. They observed that, in contrast to CE-MRA, TOF-MRA and PC-MRA were not sufficient to detect all constructed IAs and that the gold standard DSA detected all IAs. The authors postulated that this can be explained by the induced turbulent blood flow which results in signal loss in TOF-MRA and PC-MRA. This effect is overcome in CE-MRA, which uses contrast agents and images vasculature regardless of blood flow. Another study confirmed that CE-MRA is as good as DSA to detect IAs in animal models [59].

Paramagnetic objects, such as coil, may disturb the magnetic field and therefore create artifacts on MRA images. Therefore, Spilberg et al. [113] evaluated the signal overestimation, i.e., the created artifact, of CCA IAs in dogs using CE-MRA during 28 weeks after coiling. They described a gradual decay of the signal overestimation until 4 weeks post-surgery to reach a 25% decrease. Moreover, as for CTA, Dudeck et al. [79] did not report any artifact when imaging swine CCA IAs coiled with a liquid embolic agent.

MRA lacks resolution with endogenous IAs especially in small animals like rats or mice. Therefore, few studies using endogenous IA animal models used MRA (Table 1). In 2015, using MRA, Makino et al. [143] were able to detect a large aneurysm induced in a mouse cerebral artery after elastase injection in the cerebrospinal fluid. However, because of their small size, most endogenous IAs are impossible to image with MRA despite huge improvement in MRI technology with the development of 7 T or even 9.4 T MRI. Thus, most studies use MRA not to image IAs, but rather to image vascular remodeling in the circle of Willis or to determine intra-arterial hemodynamics using computational fluid dynamics (CFD) analyses [146, 156, 168].

Furthermore, as IAs with thin walls are associated with an increased risk of rupture, MRA has also been used to measure IA wall thickness in the rabbit venous pouch IA model [139]. Unfortunately, it appeared that 3 T MRI overestimates the wall thickness and that a better resolution is needed to study differences of < 0.4 mm in wall thickness. MRI can also be used to study vessel wall enhancement (VWE) after contrast agent injection in IA animal models. Indeed, in a rabbit elastase model, VWE was observed and correlated positively with the number of inflammatory cells [169]. Moreover, molecular imaging can be performed in animal models using MRA. Thus, IA wall inflammation was imaged in the rabbit elastase model after lipopolysaccharide injection using an MRI contrast agent targeting myeloperoxidases [101, 138]. In addition, Shimizu et al. [181] imaged ferumoxytol contrast agent accumulation in a rat IA wall with macrophage infiltration. Ferumoxytol is considered a true blood pool contrast agent and in addition can leak through permeable endothelium and is taken up by macrophages [201]. With targeted MRI contrast agent, it is thus possible to visualize an excess of inflammatory cells, but whether the method is sensitive enough for other potential IA instability markers remains to be proven. In this respect, it is interesting to mention that Zhang et al. [183] used a nanoplatform (zinc and iron oxide nanoparticles targeting the platelets) to target thrombus in the rabbit elastase IA model.

- **Hemodynamic imaging**

Hemodynamic imaging is a functional method that measures active changes in hemodynamic parameters, which is fundamental in studies using IA animal models as disturbed blood flow patterns control IA pathophysiology. For instance, PC-MRA is a hemodynamic imaging modality that allows for the quantitative measurement of blood flow velocity [200] and has been used in IA animal models [56, 109, 113, 181]. Doppler ultrasonography, which is another modality that measures the velocity of flow [202], was the first imaging technique enabling the study of intra-aneurysmal hemodynamics in such animal models. In 1993, Hashimoto [31] used this modality to measure blood flow velocity in a rabbit venous pouch IA model. Since then, this technique has been used in a number of IA studies to study blood flow velocity in cerebral arteries or within the IA and to check IA patency over time or after treatments [41, 42, 44, 91, 154, 176, 178].

Other hemodynamic imaging methods like CFD started to be used in animal models of IAs. CFD uses vessel geometry obtained with high-resolution 3D imaging to numerically simulate complex vascular hemodynamics [203]. Already in 2007, Kadirvel et al. [88] simulated hemodynamic forces (e.g., WSS), using CFD from 3D-DSA images in the elastase rabbit model. Interestingly, they found a correlation between altered WSS and markers of vascular remodeling. In IA animal models, CFD has been simulated from 3D-DSA [88, 98, 106, 109, 110, 123, 131–134, 144, 146] and 3D-MRA [109, 156, 181]. In the principle, CFD can also be generated from 3D-CT, but no studies using IA animal models were found. CFD simulations in IA animal models allowed for a better understanding of IA pathophysiology and participated in research for new IA treatments. Indeed, Cebral et al. [131, 134] used CFD to study hemodynamic patterns after flow diversion treatment. CFD analyses were also used to study hemodynamics in induced IAs [109, 144] and even confirmed that IA hemodynamics are similar in human IAs and elastase-induced IAs in rabbits [106]. Moreover, several studies highlighted correlations between dynamic changes in hemodynamics and vascular remodeling [88, 91, 98, 110, 132, 133, 156].

High-resolution 3D images are needed to generate CFD, which may be difficult to obtain in small animals. Therefore, it could be an option to use vascular casts created after the sacrifice of animals to re-create a precise 3D arterial geometry and therefore a precise CFD, as shown by Tutino et al., for instance [146].

- **Optical coherence tomography**

OCT is a high-resolution and less invasive optical imaging technique that uses light produced by a vascular probe (e.g., linear scanning probes or MEMS-based probes [204]) to obtain high-resolution tomography of tissues like eyes or blood vessels [205]. The near-infrared light reflects on tissue and the depth in which this reflection occurred is calculated using the delays of the back-reflected wave [205]. Indeed, OCT uses an interferometer composed of a sample arm and a reference arm to measure the interference granting a high-resolution imaging modality [204]. In 2005, Thorell et al. [67] used bench-TOP OCT on ex vivo dog-coiled surgical CCA aneurysms. They could easily identify the IA neck and coil pattern and obtained a good correlation between OCT images and histological findings. OCT was then used in vivo in induced IAs in dogs, rabbits, and rats (Table 1). OCT is mainly used for the evaluation of endovascular devices [161–163, 171, 173], i.e., IA recanalization following an incomplete coil occlusion, flow diverter malposition, or neointimal hyperplasia, which are important limitations of these treatment modalities. However, OCT does not allow the visualization of the IA form and size. Interestingly, Liu et al. [165] were able to observe internal and external elastic lamina disruption using OCT in elastase-induced IAs in rabbits. Moreover, Fries et al. [177] found OCT more sensitive as it allowed the detection of 18 residual aneurysms after flow diverter implantation in the rabbit elastase model as compared to DSA, which allowed the detection of 12 residual aneurysms only. More recently, Vardar et al. [182] showed the potential of high-frequency OCT (HF-OCT) in the rabbit elastase model to assess the correct IA occlusion after endovascular treatment as well as during follow-up imaging.

- **Fluorescence angiography**

The development of fluorescence microscopy in the beginning of the 1900s brought the possibility to observe emitted fluorescence after the excitation of a fluorophore in cultured cells or on slides [206]. Furthermore, this imaging modality can also be applied in vivo to image cells and tissues in IA animal models for instance. Indeed, in 1993, Nakatani et al. [30] used fluorescent particles to visualize blood flow in an IA rat model. More recently, a transgenic rat line expressing a green fluorescent protein specifically in endothelial cells [164, 179] was used to visualize the wall motion in IAs. Moreover, fluorescence angiography using fluorescein injection has been described to visualize blood flow and assess IA patency in rat and rabbit models [167, 170]. This imaging technique is not associated with increased mortality or morbidity and shows high contrast and sensitivity for a low-cost imaging modality. However, it is an invasive method as the artery and IA have to be dissected to be exposed to the light source.

- **Combination of imaging modalities**

A total of 44 reviewed articles (i.e., 26% of the reviewed articles) combined several imaging modalities (see Table 1). Obviously, a large portion of these studies used numerous modalities to compare different imaging techniques and research on in vivo imaging. However, other studies combined several imaging techniques to use the advantages of the different imaging modalities and acquire more information on the induced IAs. For instance, hemodynamic parameters or IA patency can be measured using Doppler ultrasonography and combined with conventional catheter angiography or MRA to image accurately the morphology and size of the IAs. Moreover, DSA being the gold standard IA imaging modality, 38 reviewed articles combined DSA with one or several other imaging techniques to visualize IA in animals. Of note, DSA is commonly used in animals during the surgical construction of IAs and in combination with CTA, MRA, or OCT after IA construction to obtain more detailed information on the morphology of the IA.

## Discussion

### Purpose of in vivo imaging of IAs in animal models

The large number of articles included in this review using surgical IA animal models reveals the paramount importance of in vivo IA imaging in such models. Indeed, to surgically construct and check the correct IA patency over time, in vivo imaging is essential. As size of surgically created IAs in animals is similar to the human ones, imaging techniques used in clinical settings can be employed in these large animal models. Moreover, surgical models are mainly used to test endovascular procedures, and in vivo imaging is necessary to assess treatment efficacy.

In contrast, only 12% of the reviewed articles use endogenous IA animal models. Indeed, in vivo imaging of IAs seems to be less often used in endogenous animal models as discussed in a recent review by Tutino et al. [15]. They showed that only 7% of studies on endogenous IAs in animals were combined with medical imaging. This lack of use can be partially explained by the fact that in vivo imaging is not essential in these studies. Indeed, most of them aim to better understand IA pathophysiology and not to test new endovascular treatment modalities. Therefore, in vivo imaging is not essential as they can directly observe the IA samples after animal euthanasia. Most studies report using (immuno) staining to characterize IA wall changes or observe artery bulging under a binocular microscope or scanning electron microscopy of circle of Willis casts. Furthermore, the lack of in vivo imaging in studies using endogenous models can be explained by an insufficient image resolution to visualize endogenously induced IAs of small size. Indeed, spatial resolution of the commonly available modalities to image IAs in animals is limited: DSA (< 0.5 mm [207]), CT (≈1 mm [7]), and MRA (1–2 mm [208]). Yet, in vivo imaging has greatly improved, and several high-resolution imaging modalities exist: 3D-DSA (0.15 mm [209]), high-resolution CTA (0.25 mm [8]), high-resolution MRA (50 µm [156]), as well as HF-OCT (10 µm [182]). Small rodents like rats and mice, which are mostly used for endogenous IA models, are often exposed to MRA and hemodynamic imaging and less frequently to conventional catheter angiography, which is mainly used in bigger animal models (Fig. 3B). As diverse IA imaging modalities are nowadays more generally available (Fig. 3C), imaging of endogenously induced IAs has become more accessible.

Despite these limitations of in vivo imaging of IAs in small animal models, there are many good reasons to perform in vivo imaging in rodents like mice or rats. Indeed, without in vivo imaging, endogenous IAs can only be studied at the sacrifice of the animal, whereas in vivo imaging permits the observation of IA size, shape, and hemodynamics at different stages during IA development. Studies monitoring IA development require endogenous animal models because surgical models do not reflect the natural IA formation and progression. Studies with follow-up imaging would lead to a better understanding of the morphological IA changes appearing before IA rupture. Such knowledge would greatly help in clinical follow-up imaging to determine whether an unruptured IA is at risk of rupture or not and whether it needs to be secured or not. Moreover, linking in vivo imaging and histology could also greatly help in this decision process. Indeed, essential changes in wall composition have been identified in ruptured human IAs when compared to unruptured IAs. Increased inflammatory cell infiltration, luminal thrombosis, and less smooth muscle cells and collagen fibers have been observed in wall of ruptured human IAs [210, 211]. The emergence of molecular imaging could allow for the in vivo visualization of these changes in the IA wall. So far, molecular imaging using targeted MRI contrast agents allowed for the visualization of the inflammation-associated tissue marker, myeloperoxidase [101, 138], macrophage infiltration using ferumoxytol [181], and thrombus using a nanoplatform targeting the platelets [183] in animal IA models. The development of other targeted MRI contrast agents would critically help to elucidate modifications in the vessel wall during IA development and prior to rupture. During MRI, the observation of VWE, which reflects a gadolinium-based contrast agent accumulation in the aneurysm wall, has been associated with an increased risk of IA rupture in human. The pathophysiological reasoning behind the occurrence of VWE is unknown, but enhanced permeability of arterial endothelium, excessive macrophage infiltration, or presence of (leaky) *vasa vasorum* have been proposed as potential mechanisms [212]. Studies using in vivo imaging and animal models of IA could help to elucidate this phenomenon, like the study of Wang et al. [169].

Therefore, in vivo IA imaging should be used more frequently in studies using endogenous animal models. Indeed, this would help to better understand morphological and hemodynamic changes of IAs during their evolution before rupture. Moreover, MRA molecular imaging allows for the observation of in vivo wall modifications. Thus, it is essential that in vivo imaging continues to improve to obtain images of small IAs in endogenous animal models at sufficient resolution.

### Comparison of the IA in vivo imaging techniques

In vivo imaging is important for studies using surgical and endogenous IA animal models, and many imaging techniques are now available (Fig. 3C). All imaging modalities have advantages and disadvantages (Table 1), and it is essential to choose the most appropriate modality.

All imaging modalities do not provide the same resolution, which is a first consideration. Based on human data, conventional MRA and CTA exhibit an insufficient resolution for IAs having a diameter < 3 mm [7]. Therefore, these techniques are not appropriate for endogenously induced IAs in small animals which require high-resolution imaging. DSA is the gold standard technique as it displays a high resolution that can further be increased with 3D-DSA [8]. The resolution of CTA seems to be increased when combined with a flat detector [102]. MRA resolution can be improved using a higher magnetic field (7 T or even 9.4 T), which has been shown to allow for accurate imaging of the rat circle of Willis [156, 168, 174]. More recently, HF-OCT showed a great potential to assess appropriate treatment of IAs, thanks to a very high-spatial resolution close to 10 μm [182]. CFD uses high-resolution 3D imaging to simulate the flow at every position in the IA and adjacent arteries. In comparison, Doppler ultrasonography measures the average blood flow velocity for the entire IA, which is less precise for IA studies. Human studies comparing both techniques show that WSS measured by Doppler ultrasonography is consistently smaller compared to CFD simulations [213].

The choice of the imaging modality should obviously take the invasiveness of the procedure into consideration. Indeed, when several imaging modalities allow for IA visualization at sufficient resolution for the goal of the study, the less invasive technique should be selected. DSA, despite being the gold standard technique, remains the more invasive modality. However, the use of a venous instead of an arterial catheter decreases DSA invasiveness. Fluorescence angiography is also invasive, as it requires artery dissection. CTA is a less invasive technique as the ionizing radiation is lower and as the contrast agent is injected through a venous catheter. Finally, MRA and OCT are the less invasive in vivo imaging modalities for IAs as they do not require ionizing radiation nor contrast agent injection except for CE-MRA that requires a venous contrast agent injection. Long-term and repetitive studies should obviously use the less invasive IA in vivo imaging modality. Doerfler et al. [18] showed that induced IAs in the rabbit elastase model can be serially imaged during a long-term study using i.a. DSA, i.v. DSA, CTA, and MRA. Therefore, the less invasive CTA and MRA modalities should be preferred over the more invasive techniques.

IA patency, with or without endovascular treatment, can be evaluated using different in vivo imaging modalities. DSA, CTA, MRA, and Doppler ultrasonography are routinely used, and OCT, which is a more recent high-resolution technique, is very efficient to assess IA patency accurately [182]. As discussed above, DSA is the more invasive technique and should be avoided when possible. OCT being non-invasive and displaying a high resolution should be preferred. However, this imaging modality does not allow for global morphology visualization of IAs and may be combined with another imaging modality. The presence of artifacts in some imaging modalities due to endovascular treatments should also be considered. Indeed, artifacts in presence of clips, stent, or coils can be observed in CT and MRA [192]. Metal artifact reduction software are available for clinical CT [214]; Yuki et al. [147] successfully decreased CT stent artifacts in a swine model of IAs. Moreover, Spilberg et al. [113] observed a decay in MRA artifact until 4 weeks post-surgery, which could be linked to IA thrombus modifications.

This review did not discuss 4D imaging because only 2 reviewed studies used time-resolved 4D imaging, which combined sequentially obtained 3D images [108, 109]. However, such imaging techniques are known to significantly improve imaging in clinic. For instance, 4D-DSA could lead to a voxel volume of 0.008mm^3^ [215]. Temporal resolution is an important parameter to consider in time-resolved imaging, as it will determine the capability of the imaging modality to distinguish fast physiological temporal processes.

### Limitations of the study

Despite a careful database search following the PRISMA guidelines and using precise MeSH terms and additional hand searches, this systematic review might have missed some studies using in vivo imaging in IA animal models. Therefore, we cannot exclude a slight bias in the distribution of the different IA animal models and imaging modalities.

## Conclusion

In vivo imaging of IAs has tremendously improved in recent years and should be used more frequently in IA animal models. However, all imaging techniques have advantages and disadvantages, and the most appropriate imaging modality should be chosen. The imaging resolution and invasiveness should be considered with respect to the goal of the study. In particular, studies aiming to test endovascular treatment should consider ability to assess IA patency of the imaging modality and the presence of potential metal artifacts. Research to improve imaging modalities should continue, in particular in the field of molecular imaging to better understand IA physiopathology.

## References

[1] Brisman JL, Song JK, Newell DW (2006). Cerebral aneurysms. N Engl J Med.

[2] Diagbouga MR, Morel S, Bijlenga P, Kwak BR (2018). Role of hemodynamics in initiation/growth of intracranial aneurysms. Eur J Clin Invest.

[3] Texakalidis P, Sweid A, Mouchtouris N, Peterson EC, Sioka C, Rangel-Castilla L (2019). Aneurysm formation, growth, and rupture: the biology and physics of cerebral aneurysms. World Neurosurg.

[4] Hackenberg KAM, Hänggi D, Etminan N (2018). Unruptured intracranial aneurysms. Stroke.

[5] Lawton MT, Vates GE (2017). Subarachnoid hemorrhage. N Engl J Med.

[6] Taufique Z, May T, Meyers E, Falo C, Mayer SA, Agarwal S (2016). Predictors of poor quality of life 1 year after subarachnoid hemorrhage. Neurosurgery.

[7] Turan N, Heider RA, Roy AK, Miller BA, Mullins ME, Barrow DL (2018). Current perspectives in imaging modalities for the assessment of unruptured intracranial aneurysms: a comparative analysis and review. World Neurosurg.

[8] Maupu C, Lebas H, Boulaftali Y (2022). Imaging modalities for intracranial aneurysm: more than meets the eye. Front Cardiovasc Med.

[9] Bijlenga P, Gondar R, Schilling S, Morel S, Hirsch S, Cuony J (2017). PHASES score for the management of intracranial aneurysm: a cross-sectional population-based retrospective study. Stroke.

[10] Backes D, Rinkel GJE, Greving JP, Velthuis BK, Murayama Y, Takao H (2017). ELAPSS score for prediction of risk of growth of unruptured intracranial aneurysms. Neurology.

[11] Etminan N, Brown RD, Beseoglu K, Juvela S, Raymond J, Morita A (2015). The unruptured intracranial aneurysm treatment score: a multidisciplinary consensus. Neurology.

[12] Massoud TF, Guglielmi G, Ji C, Viñuela F, Duckwiler GR (1994). Experimental saccular aneurysms. I. Review of surgically-constructed models and their laboratory applications. Neuroradiology.

[13] German WJ, Black SP (1954). Experimental production of carotid aneurysms. N Engl J Med.

[14] Altes TA, Cloft HJ, Short JG, DeGast A, Do HM, Helm GA (2000). 1999 ARRS Executive Council Award. Creation of saccular aneurysms in the rabbit: a model suitable for testing endovascular devices. American Roentgen Ray Society. AJR Am J Roentgenol.

[15] Tutino VM, Rajabzadeh-Oghaz H, Veeturi SS, Poppenberg KE, Waqas M, Mandelbaum M (2021). Endogenous animal models of intracranial aneurysm development: a review. Neurosurg Rev.

[16] Etminan N, Rinkel GJ (2016). Unruptured intracranial aneurysms: development, rupture and preventive management. Nat Rev Neurol.

[17] Bouzeghrane F, Naggara O, Kallmes DF, Berenstein A, Raymond J (2010). In vivo experimental intracranial aneurysm models: a systematic review. AJNR Am J Neuroradiol.

[18] Doerfler A, Becker W, Wanke I, Goericke S, Oezkan N, Forsting M (2004). Multimodal imaging in the elastase-induced aneurysm model in rabbits: a comparative study using serial DSA, MRA and CTA. Rofo.

[19] Page MJ, McKenzie JE, Bossuyt PM, Boutron I, Hoffmann TC, Mulrow CD (2021). The PRISMA 2020 statement: an updated guideline for reporting systematic reviews. BMJ.

[20] Black SP, German WJ (1960). Observations on the relationship between the volume and the size of the orifice of experimental aneurysms. J Neurosurg.

[21] Roy VC, Sundt TM, Murphey F (1970). Experimental subarachnoid hemorrhage: a study for spasm with the production of aneurysms. Stroke.

[22] Cares HL, Hale JR, Montgomery DB, Richter HA, Sweet WH (1973). Laboratory experience with a magnetically guided intravascular catheter system. J Neurosurg.

[23] Maira G, Mohr G, Panisset A, Hardy J (1979). Laser photocoagulation for treatment of experimental aneurysms. J Microsurg.

[24] Nagata I, Handa H, Hashimoto N (1979). Experimentally induced cerebral aneurysms in rats: part IV–cerebral angiography. Surg Neurol.

[25] Sandor T, Utsonomyia R, Rumbaugh C, Sridhar B (1980). On the feasibility of densitometric assessment of cerebral aneurysms. Int J Biomed Comput.

[26] O'Reilly GV, Utsunomiya R, Rumbaugh CL, Colucci VM (1981). Experimental arterial aneurysms:modification of the production technique. J Microsurg.

[27] Hashimoto N, Handa H, Nagata I, Hazama F (1984). Animal model of cerebral aneurysms: pathology and pathogenesis of induced cerebral aneurysms in rats. Neurol Res.

[28] Hashimoto N, Kim C, Kikuchi H, Kojima M, Kang Y, Hazama F (1987). Experimental induction of cerebral aneurysms in monkeys. J Neurosurg.

[29] Forrest MD, O'Reilly GV (1989). Production of experimental aneurysms at a surgically created arterial bifurcation. AJNR Am J Neuroradiol.

[30] Nakatani H, Hashimoto N, Kikuchi H, Yamaguchi S, Niimi H (1993). In vivo flow visualization of induced saccular cerebral aneurysms in rats. Acta Neurochir (Wien).

[31] Hashimoto T (1993). Flow velocity studies in vein pouch model aneurysms. Neurol Res.

[32] Graves VB, Ahuja A, Strother CM, Rappe AH (1993). Canine model of terminal arterial aneurysm. AJNR Am J Neuroradiol.

[33] Massoud TF, Ji C, Guglielmi G, Viñuela F, Robert J (1994). Experimental models of bifurcation and terminal aneurysms: construction techniques in swine. AJNR Am J Neuroradiol.

[34] Geremia G, Haklin M, Brennecke L (1994). Embolization of experimentally created aneurysms with intravascular stent devices. AJNR Am J Neuroradiol.

[35] Guglielmi G, Ji C, Massoud TF, Kurata A, Lownie SP, Viñuela F (1994). Experimental saccular aneurysms. II. A new model in swine Neuroradiology.

[36] Wakhloo AK, Schellhammer F, de Vries J, Haberstroh J, Schumacher M (1994). Self-expanding and balloon-expandable stents in the treatment of carotid aneurysms: an experimental study in a canine model. AJNR Am J Neuroradiol.

[37] Cawley CM, Dawson RC, Shengelaia G, Bonner G, Barrow DL, Colohan AR (1996). Arterial saccular aneurysm model in the rabbit. AJNR Am J Neuroradiol.

[38] Kirse DJ, Flock S, Teo C, Rahman S, Mrak R (1996). Construction of a vein-pouch aneurysm at a surgically created carotid bifurcation in the rat. Microsurgery.

[39] Spetzger U, Reul J, Weis J, Bertalanffy H, Thron A, Gilsbach JM (1996). Microsurgically produced bifurcation aneurysms in a rabbit model for endovascular coil embolization. J Neurosurg.

[40] Bavinzski G, Al-Schameri A, Killer M, Schwendenwein I, Gruber A, Saringer W (1998). Experimental bifurcation aneurysm: a model for in vivo evaluation of endovascular techniques. Minim Invasive Neurosurg.

[41] Fukui K, Negoro M, Keino H, Yoshida J (1998). Experimental creation of fusiform carotid artery aneurysms using vein grafts in rats. Neurosurgery.

[42] Kallmes DF, Altes TA, Vincent DA, Cloft HJ, Do HM, Jensen ME (1999). Experimental side-wall aneurysms: a natural history study. Neuroradiology.

[43] Kallmes DF, Helm GA, Hudson SB, Altes TA, Do HM, Mandell JW (1999). Histologic evaluation of platinum coil embolization in an aneurysm model in rabbits. Radiology.

[44] Ujiie H, Tachibana H, Hiramatsu O, Hazel AL, Matsumoto T, Ogasawara Y (1999). Effects of size and shape (aspect ratio) on the hemodynamics of saccular aneurysms: a possible index for surgical treatment of intracranial aneurysms. Neurosurgery.

[45] Cloft HJ, Altes TA, Marx WF, Raible RJ, Hudson SB, Helm GA (1999). Endovascular creation of an in vivo bifurcation aneurysm model in rabbits. Radiology.

[46] Murayama Y, Viñuela F, Suzuki Y, Akiba Y, Ulihoa A, Duckwiler GR (1999). Development of the biologically active Guglielmi detachable coil for the treatment of cerebral aneurysms. Part II: an experimental study in a swine aneurysm model. AJNR Am J Neuroradiol.

[47] Murayama Y, Viñuela F, Tateshima S, Song JK, Gonzalez NR, Wallace MP (2001). Bioabsorbable polymeric material coils for embolization of intracranial aneurysms: a preliminary experimental study. J Neurosurg.

[48] Short JG, Fujiwara NH, Marx WF, Helm GA, Cloft HJ, Kallmes DF (2001). Elastase-induced saccular aneurysms in rabbits: comparison of geometric features with those of human aneurysms. AJNR Am J Neuroradiol.

[49] de Gast AN, Altes TA, Marx WF, Do HM, Helm GA, Kallmes DF (2001). Transforming growth factor beta-coated platinum coils for endovascular treatment of aneurysms: an animal study. Neurosurgery.

[50] Fujiwara NH, Cloft HJ, Marx WF, Short JG, Jensen ME, Kallmes DF (2001). Serial angiography in an elastase-induced aneurysm model in rabbits: evidence for progressive aneurysm enlargement after creation. AJNR Am J Neuroradiol.

[51] Raymond J, Berthelet F, Desfaits AC, Salazkin I, Roy D (2002). Cyanoacrylate embolization of experimental aneurysms. AJNR Am J Neuroradiol.

[52] Kallmes DF, Fujiwara NH, Berr SS, Helm GA, Cloft HJ (2002). Elastase-induced saccular aneurysms in rabbits: a dose-escalation study. AJNR Am J Neuroradiol.

[53] Fujiwara NH, Kallmes DF (2002). Healing response in elastase-induced rabbit aneurysms after embolization with a new platinum coil system. AJNR Am J Neuroradiol.

[54] Raymond J, Salazkin I, Georganos S, Guilbert F, Desfaits AC, Gevry G (2002). Endovascular treatment of experimental wide neck aneurysms: comparison of results using coils or cyanoacrylate with the assistance of an aneurysm neck bridge device. AJNR Am J Neuroradiol.

[55] Kallmes DF, Fujiwara NH (2002). New expandable hydrogel-platinum coil hybrid device for aneurysm embolization. AJNR Am J Neuroradiol.

[56] Krings T, Hans FJ, Möller-Hartmann W, Thiex R, Brunn A, Scherer K (2002). Time-of-flight-, phase contrast and contrast enhanced magnetic resonance angiography for pre-interventional determination of aneurysm size, configuration, and neck morphology in an aneurysm model in rabbits. Neurosci Lett.

[57] Kallmes DF, Fujiwara NH, Yuen D, Dai D, Li ST (2003). A collagen-based coil for embolization of saccular aneurysms in a New Zealand White rabbit model. AJNR Am J Neuroradiol.

[58] Raymond J, Metcalfe A, Desfaits AC, Ribourtout E, Salazkin I, Gilmartin K (2003). Alginate for endovascular treatment of aneurysms and local growth factor delivery. AJNR Am J Neuroradiol.

[59] Krings T, Möller-Hartmann W, Hans FJ, Thiex R, Brunn A, Scherer K (2003). A refined method for creating saccular aneurysms in the rabbit. Neuroradiology.

[60] Murayama Y, Tateshima S, Gonzalez NR, Vinuela F (2003). Matrix and bioabsorbable polymeric coils accelerate healing of intracranial aneurysms: long-term experimental study. Stroke.

[61] Möller-Hartmann W, Krings T, Stein KP, Dreeskamp A, Meetz A, Thiex R (2003). Aberrant origin of the superior thyroid artery and the tracheoesophageal branch from the common carotid artery: a source of failure in elastase-induced aneurysms in rabbits. AJR Am J Roentgenol.

[62] Thiex R, Hans FJ, Krings T, Möller-Hartmann W, Brunn A, Scherer K (2004). Haemorrhagic tracheal necrosis as a lethal complication of an aneurysm model in rabbits via endoluminal incubation with elastase. Acta Neurochir (Wien).

[63] Raymond J, Salazkin I, Metcalfe A, Robledo O, Gevry G, Roy D (2004). Lingual artery bifurcation aneurysms for training and evaluation of neurovascular devices. AJNR Am J Neuroradiol.

[64] Yoshino Y, Niimi Y, Song JK, Silane M, Berenstein A (2004). Endovascular treatment of intracranial aneurysms: comparative evaluation in a terminal bifurcation aneurysm model in dogs. J Neurosurg.

[65] Hoh BL, Rabinov JD, Pryor JC, Ogilvy CS (2004). A modified technique for using elastase to create saccular aneurysms in animals that histologically and hemodynamically resemble aneurysms in human. Acta Neurochir (Wien).

[66] Seong J, Sadasivan C, Onizuka M, Gounis MJ, Christian F, Miskolczi L (2005). Morphology of elastase-induced cerebral aneurysm model in rabbit and rapid prototyping of elastomeric transparent replicas. Biorheology.

[67] Thorell WE, Chow MM, Prayson RA, Shure MA, Jeon SW, Huang D (2005). Optical coherence tomography: a new method to assess aneurysm healing. J Neurosurg.

[68] Shin YS, Niimi Y, Yoshino Y, Song JK, Silane M, Berenstein A (2005). Creation of four experimental aneurysms with different hemodynamics in one dog. AJNR Am J Neuroradiol.

[69] Ding YH, Dai D, Lewis DA, Danielson MA, Kadirvel R, Mandrekar JN (2005). Can neck size in elastase-induced aneurysms be controlled? A prospective study. AJNR Am J Neuroradiol.

[70] Raymond J, Ogoudikpe C, Salazkin I, Metcalfe A, Gevry G, Chagnon M (2005). Endovascular treatment of aneurysms: gene expression of neointimal cells recruited on the embolic agent and evolution with recurrence in an experimental model. J Vasc Interv Radiol.

[71] Grunwald IQ, Romeike BF, Roth C, Struffert T, Eymann R, Reith W (2005). Anticoagulation regimes and their influence on the occlusion rate of aneurysms: an experimental study in rabbits. Neurosurgery.

[72] Boulos AS, Deshaies EM, Fessler RD, Aketa S, Standard S, Miskolczi L (2005). A triple bifurcation aneurysm model for evaluating complex endovascular therapies in dogs. J Neurosurg.

[73] Ding YH, Dai D, Lewis DA, Danielson MA, Kadirvel R, Cloft HJ (2006). Long-term patency of elastase-induced aneurysm model in rabbits. AJNR Am J Neuroradiol.

[74] Sadasivan C, Lieber BB, Cesar L, Miskolczi L, Seong J, Wakhloo AK (2006). Angiographic assessment of the performance of flow divertors to treat cerebral aneurysms. Conf Proc IEEE Eng Med Biol Soc.

[75] Onizuka M, Miskolczi L, Gounis MJ, Seong J, Lieber BB, Wakhloo AK (2006). Elastase-induced aneurysms in rabbits: effect of postconstruction geometry on final size. AJNR Am J Neuroradiol.

[76] Ding YH, Danielson MA, Kadirvel R, Dai D, Lewis DA, Cloft HJ (2006). Modified technique to create morphologically reproducible elastase-induced aneurysms in rabbits. Neuroradiology.

[77] Acar F, Men S, Tayfur V, Yilmaz O, Erbayraktar S, Metin Güner E (2006). In vivo intraaneurysmal pressure measurements in experimental lateral wall aneurysms before and after onyx embolization. Surg Neurol.

[78] Ding YH, Dai D, Danielson MA, Kadirvel R, Lewis DA, Cloft HJ (2006). Intra-arterial digital subtraction angiography through the ear artery for experimental aneurysm imaging. AJNR Am J Neuroradiol.

[79] Dudeck O, Jordan O, Hoffmann KT, Okuducu AF, Husmann I, Kreuzer-Nagy T (2006). Embolization of experimental wide-necked aneurysms with iodine-containing polyvinyl alcohol solubilized in a low-angiotoxicity solvent. AJNR Am J Neuroradiol.

[80] Dudeck O, Okuducu AF, Jordan O, Tesmer K, Pech M, Weigang E (2006). Volume changes of experimental carotid sidewall aneurysms due to embolization with liquid embolic agents: a multidetector CT angiography study. Cardiovasc Intervent Radiol.

[81] Frösen J, Marjamaa J, Myllärniemi M, Abo-Ramadan U, Tulamo R, Niemelä M (2006). Contribution of mural and bone marrow-derived neointimal cells to thrombus organization and wall remodeling in a microsurgical murine saccular aneurysm model. Neurosurgery.

[82] Dai D, Ding YH, Lewis DA, Kallmes DF (2006). A proposed ordinal scale for grading histology in elastase-induced, saccular aneurysms. AJNR Am J Neuroradiol.

[83] Dai D, Ding YH, Danielson MA, Kadirvel R, Hunter LW, Zhan WZ (2007). Endovascular treatment of experimental aneurysms by use of fibroblast-coated platinum coils: an angiographic and histopathologic study. Stroke.

[84] Song JK, Niimi Y, Yoshino Y, Khoyama S, Berenstein A (2007). Assessment of Matrix coils in a canine model of a large bifurcation aneurysm. Neuroradiology.

[85] Ding YH, Dai D, Danielson MA, Kadirvel R, Lewis DA, Cloft HJ (2007). Control of aneurysm volume by adjusting the position of ligation during creation of elastase-induced aneurysms: a prospective study. AJNR Am J Neuroradiol.

[86] Kallmes DF, Ding YH, Dai D, Kadirvel R, Lewis DA, Cloft HJ (2007). A new endoluminal, flow-disrupting device for treatment of saccular aneurysms. Stroke.

[87] Ahlhelm F, Roth C, Kaufmann R, Schulte-Altedorneburg G, Romeike BF, Reith W (2007). Treatment of wide-necked intracranial aneurysms with a novel self-expanding two-zonal endovascular stent device. Neuroradiology.

[88] Kadirvel R, Ding YH, Dai D, Zakaria H, Robertson AM, Danielson MA (2007). The influence of hemodynamic forces on biomarkers in the walls of elastase-induced aneurysms in rabbits. Neuroradiology.

[89] Turk AS, Luty CM, Carr-Brendel V, Polyakov I, Consigny D, Grinde J (2008). Angiographic and histological comparison of canine bifurcation aneurysms treated with first generation matrix and standard GDC coils. Neuroradiology.

[90] Tsumoto T, Song JK, Niimi Y, Berenstein A (2008). Interval change in size of venous pouch canine bifurcation aneurysms over a 10-month period. AJNR Am J Neuroradiol.

[91] Gao L, Hoi Y, Swartz DD, Kolega J, Siddiqui A, Meng H (2008). Nascent aneurysm formation at the basilar terminus induced by hemodynamics. Stroke.

[92] Struffert T, Roth C, Romeike B, Grunwald IO, Reith W (2008). Onyx in an experimental aneurysm model: histological and angiographic results. J Neurosurg.

[93] Arends J, Perkins KD, Zhang J, Polyakov I, Lee E (2008). A new technique for the surgical creation of aneurysms in an in vivo tortuous vessel model to test neurovascular devices. J Invest Surg.

[94] Berenstein A, Song JK, Tsumoto T, Namba K, Niimi Y (2009). Treatment of experimental aneurysms with an embolic-containing device and liquid embolic agent: feasibility and angiographic and histological results. Neurosurgery.

[95] Sadasivan C, Cesar L, Seong J, Rakian A, Hao Q, Tio FO (2009). An original flow diversion device for the treatment of intracranial aneurysms: evaluation in the rabbit elastase-induced model. Stroke.

[96] Marjamaa J, Tulamo R, Frösen J, Abo-Ramadan U, Hernesniemi JA, Niemelä MR (2009). Occlusion of neck remnant in experimental rat aneurysms after treatment with platinum- or polyglycolic-polylactic acid-coated coils. Surg Neurol.

[97] Tsumoto T, Niimi Y, Berenstein A (2009). Evaluation of the new HydroSoft coil in a canine model of bifurcation aneurysm. Laboratory investigation J Neurosurg.

[98] Wang Z, Kolega J, Hoi Y, Gao L, Swartz DD, Levy EI (2009). Molecular alterations associated with aneurysmal remodeling are localized in the high hemodynamic stress region of a created carotid bifurcation. Neurosurgery.

[99] Killer M, Kallmes DF, McCoy MR, Ding YH, Shum JC, Cruise GM (2009). Angiographic and histologic comparison of experimental aneurysms embolized with hydrogel filaments. AJNR Am J Neuroradiol.

[100] Takao H, Murayama Y, Yuki I, Ishibashi T, Ebara M, Irie K (2009). Endovascular treatment of experimental aneurysms using a combination of thermoreversible gelation polymer and protection devices: feasibility study. Neurosurgery.

[101] DeLeo MJ, Gounis MJ, Hong B, Ford JC, Wakhloo AK, Bogdanov AA (2009). Carotid artery brain aneurysm model: in vivo molecular enzyme-specific MR imaging of active inflammation in a pilot study. Radiology.

[102] Struffert T, Doelken M, Adamek E, Schwarz M, Engelhorn T, Kloska S (2010). Flat-detector computed tomography with intravenous contrast material application in experimental aneurysms: comparison with multislice CT and conventional angiography. Acta Radiol.

[103] Reinges MH, Krings T, Drexler AY, Ludolph A, Sellhaus B, Bovi M (2010). Bare, bio-active and hydrogel-coated coils for endovascular treatment of experimentally induced aneurysms. Long-term histological and scanning electron microscopy results. Interv Neuroradiol.

[104] Killer M, Kallmes D, Jones R, Ding Y, Vestal M, Hauser T (2010). Long-term angiographic and histological results of a new hydrogel-containing filling coil in experimental rabbit aneurysms. Minim Invasive Neurosurg.

[105] Sherif C, Marbacher S, Erhardt S, Fandino J (2011). Improved microsurgical creation of venous pouch arterial bifurcation aneurysms in rabbits. AJNR Am J Neuroradiol.

[106] Zeng Z, Kallmes DF, Durka MJ, Ding Y, Lewis D, Kadirvel R (2011). Hemodynamics and anatomy of elastase-induced rabbit aneurysm models: similarity to human cerebral aneurysms?. AJNR Am J Neuroradiol.

[107] Marbacher S, Erhardt S, Schläppi JA, Coluccia D, Remonda L, Fandino J (2011). Complex bilobular, bisaccular, and broad-neck microsurgical aneurysm formation in the rabbit bifurcation model for the study of upcoming endovascular techniques. AJNR Am J Neuroradiol.

[108] Gupta R, Mehndiratta A, Mitha AP, Grasruck M, Leidecker C, Ogilvy C (2011). Temporal resolution of dynamic angiography using flat panel volume CT: in vivo evaluation of time-dependent vascular pathologies. AJNR Am J Neuroradiol.

[109] Jiang J, Johnson K, Valen-Sendstad K, Mardal KA, Wieben O, Strother C (2011). Flow characteristics in a canine aneurysm model: a comparison of 4D accelerated phase-contrast MR measurements and computational fluid dynamics simulations. Med Phys.

[110] Kolega J, Gao L, Mandelbaum M, Mocco J, Siddiqui AH, Natarajan SK (2011). Cellular and molecular responses of the basilar terminus to hemodynamics during intracranial aneurysm initiation in a rabbit model. J Vasc Res.

[111] Cai J, He C, Yuan F, Chen L, Ling F (2012). A novel haemodynamic cerebral aneurysm model of rats with normal blood pressure. J Clin Neurosci.

[112] Ysuda R, Strother CM, Aagaard-Kienitz B, Pulfer K, Consigny D (2012). A large and giant bifurcation aneurysm model in canines: proof of feasibility. AJNR Am J Neuroradiol.

[113] Spilberg G, Carniato SL, King RM, van der Bom IM, Mehra M, Walvick RP (2012). Temporal evolution of susceptibility artifacts from coiled aneurysms on MR angiography: an in vivo canine study. AJNR Am J Neuroradiol.

[114] Darsaut TE, Bing F, Salazkin I, Gevry G, Raymond J (2012). Flow diverters failing to occlude experimental bifurcation or curved sidewall aneurysms: an in vivo study in canines. J Neurosurg.

[115] Darsaut TE, Bing F, Salazkin I, Gevry G, Raymond J (2012). Flow diverters can occlude aneurysms and preserve arterial branches: a new experimental model. AJNR Am J Neuroradiol.

[116] Marbacher S, Tastan I, Neuschmelting V, Erhardt S, Coluccia D, Sherif C (2012). Long-term patency of complex bilobular, bisaccular, and broad-neck aneurysms in the rabbit microsurgical venous pouch bifurcation model. Neurol Res.

[117] Mühlenbruch G, Nikoubashman O, Steffen B, Dadak M, Palmowski M, Wiesmann M (2013). Endovascular broad-neck aneurysm creation in a porcine model using a vascular plug. Cardiovasc Intervent Radiol.

[118] Struffert T, Ott S, Kowarschik M, Bender F, Adamek E, Engelhorn T (2013). Measurement of quantifiable parameters by time-density curves in the elastase-induced aneurysm model: first results in the comparison of a flow diverter and a conventional aneurysm stent. Eur Radiol.

[119] Raymond J, Darsaut TE, Kotowski M, Makoyeva A, Gevry G, Berthelet F (2013). Thrombosis heralding aneurysmal rupture: an exploration of potential mechanisms in a novel giant swine aneurysm model. AJNR Am J Neuroradiol.

[120] Wang JB, Zhou B, Gu XL, Li MH, Gu BX, Wang W (2013). Treatment of a canine carotid artery aneurysm model with a biodegradable nanofiber-covered stent: a prospective pilot study. Neurol India.

[121] Turk A, Turner RD, Tateshima S, Fiorella D, Jang KS, Chaudry I (2013). Novel aneurysm neck reconstruction device: initial experience in an experimental preclinical bifurcation aneurysm model. J Neurointerv Surg.

[122] Brennecka CR, Preul MC, Becker TA, Vernon BL (2013). In vivo embolization of lateral wall aneurysms in canines using the liquid-to-solid gelling PPODA-QT polymer system: 6-month pilot study. J Neurosurg.

[123] Huang Q, Xu J, Cheng J, Wang S, Wang K, Liu JM (2013). Hemodynamic changes by flow diverters in rabbit aneurysm models: a computational fluid dynamic study based on micro-computed tomography reconstruction. Stroke.

[124] Wang Y, Ma C, Xu N, Xu K, Wang H, Yu J (2013). An improved elastase-based method to create a saccular aneurysm rabbit model. Br J Neurosurg.

[125] Kühn AL, Roth C, Romeike B, Grunwald IQ (2014). Treatment of elastase-induced intracranial aneurysms in New Zealand white rabbits by use of a novel neurovascular embolization stent device. Neuroradiology.

[126] Simgen A, Ley D, Roth C, Yilmaz U, Körner H, Mühl-Benninghaus R (2014). Evaluation of a newly designed flow diverter for the treatment of intracranial aneurysms in an elastase-induced aneurysm model, in New Zealand white rabbits. Neuroradiology.

[127] Mitome-Mishima Y, Yamamoto M, Yatomi K, Nonaka S, Miyamoto N, Urabe T, et al. (2014) Endothelial cell proliferation in swine experimental aneurysm after coil embolization. PLoS One 9: e89047. 10.1371/journal.pone.0089047 10.1371/journal.pone.0089047 PMC3925181 24551215

[128] Liu Y, Zhang Y, Dai D, Xu Z (2014). Expression of NF-κB, MCP-1 and MMP-9 in a cerebral aneurysm rabbit model. Can J Neurol Sci.

[129] Donzelli R, Mariniello G, Vitelli M, Capone C, Sgulò F, Dones F (2014). Reconstruction of artery wall in experimental giant aneurysms. J Neurosurg Sci.

[130] Erhardt S, Marbacher S, Neuschmelting V, Coluccia D, Remonda L, Fandino J (2014). Comparison between routine cylindrical cerebral aneurysm volume approximation and three-dimensional volume measurements in experimental aneurysms. Neurol Res.

[131] Cebral JR, Mut F, Raschi M, Hodis S, Ding YH, Erickson BJ (2014). Analysis of hemodynamics and aneurysm occlusion after flow-diverting treatment in rabbit models. AJNR Am J Neuroradiol.

[132] Wang J, Tan HQ, Zhu YQ, Li MH, Li ZZ, Yan L (2014). Complex hemodynamic insult in combination with wall degeneration at the apex of an arterial bifurcation contributes to generation of nascent aneurysms in a canine model. AJNR Am J Neuroradiol.

[133] Zhu YQ, Li MH, Yan L, Tan HQ, Cheng YS (2014). Arterial wall degeneration plus hemodynamic insult cause arterial wall remodeling and nascent aneurysm formation at specific sites in dogs. J Neuropathol Exp Neurol.

[134] Cebral JR, Mut F, Raschi M, Ding YH, Kadirvel R, Kallmes D (2014). Strategy for analysis of flow diverting devices based on multi-modality image-based modeling. Int J Numer Method Biomed Eng.

[135] Darsaut TE, Bing F, Makoyeva A, Gevry G, Salazkin I, Raymond J (2014). Flow diversion of giant curved sidewall and bifurcation experimental aneurysms with very-low-porosity devices. World Neurosurg.

[136] Marbacher S, Frösén J, Marjamaa J, Anisimov A, Honkanen P, von Gunten M (2014). Intraluminal cell transplantation prevents growth and rupture in a model of rupture-prone saccular aneurysms. Stroke.

[137] Marbacher S, Marjamaa J, Bradacova K, von Gunten M, Honkanen P, Abo-Ramadan U (2014). Loss of mural cells leads to wall degeneration, aneurysm growth, and eventual rupture in a rat aneurysm model. Stroke.

[138] Gounis MJ, van der Bom IM, Wakhloo AK, Zheng S, Chueh JY, Kühn AL (2015). MR imaging of myeloperoxidase activity in a model of the inflamed aneurysm wall. AJNR Am J Neuroradiol.

[139] Sherif C, Kleinpeter G, Loyoddin M, Mach G, Plasenzotti R, Haider T (2015). Aneurysm wall thickness measurements of experimental aneurysms: in vivo high-field MR imaging versus direct microscopy. Acta Neurochir Suppl.

[140] Chavan R, Pons S, Gupta V, Hui D, Bose A (2015). Safety and performance of the Penumbra Liberty stent system in a rabbit aneurysm model. J Neurointerv Surg.

[141] Krähenbühl AK, Gralla J, Abu-Isa J, Mordasini P, Widmer HR, Raabe A (2015). High-flow venous pouch aneurysm in the rabbit carotid artery: a model for large aneurysms. Interv Neuroradiol.

[142] Ott S, Gölitz P, Adamek E, Royalty K, Doerfler A, Struffert T (2015). Flat-detector computed tomography evaluation in an experimental animal aneurysm model after endovascular treatment: a pilot study. Interv Neuroradiol.

[143] Makino H, Hokamura K, Natsume T, Kimura T, Kamio Y, Magata Y (2015). Successful serial imaging of the mouse cerebral arteries using conventional 3-T magnetic resonance imaging. J Cereb Blood Flow Metab.

[144] Jiang YZ, Lan Q, Wang QH, Wang SZ, Lu H, Wu WJ (2015). Creation of experimental aneurysms at a surgically created arterial confluence. Eur Rev Med Pharmacol Sci.

[145] Puffer C, Dai D, Ding YH, Cebral J, Kallmes D, Kadirvel R (2015). Gene expression comparison of flow diversion and coiling in an experimental aneurysm model. J Neurointerv Surg.

[146] Tutino VM, Liaw N, Spernyak JA, Ionita CN, Siddiqui AH, Kolega J (2016). Assessment of vascular geometry for bilateral carotid artery ligation to induce early basilar terminus aneurysmal remodeling in rats. Curr Neurovasc Res.

[147] Yuki I, Kambayashi Y, Ikemura A, Abe Y, Kan I, Mohamed A (2016). High-resolution C-arm CT and metal artifact reduction software: a novel imaging modality for analyzing aneurysms treated with stent-assisted coil embolization. AJNR Am J Neuroradiol.

[148] Rouchaud A, Brinjikji W, Ding YH, Dai D, Zhu YQ, Cloft HJ (2016). Evaluation of the angiographic grading scale in aneurysms treated with the WEB device in 80 rabbits: correlation with histologic evaluation. AJNR Am J Neuroradiol.

[149] Ding Y, Dai D, Kallmes DF, Schroeder D, Kealey CP, Gupta V (2016). Preclinical testing of a novel thin film nitinol flow-diversion stent in a rabbit elastase aneurysm model. AJNR Am J Neuroradiol.

[150] Miura Y, Tanemura H, Fujimoto M, Hamada K, Miyamoto K, Toma N (2016). Aneurysm organization effects of gellan sulfate core platinum coil with Tenascin-C in a simulated clinical setting and the possible mechanism. J Stroke Cerebrovasc Dis.

[151] Ding YH, Dai D, Schroeder D, Kadirvel R, Kallmes DF (2016). Experimental testing of the dual-layer Woven EndoBridge device using an elastase-induced aneurysm model in rabbits. Interv Neuroradiol.

[152] Brinjikji W, Yong Hong D, Dai D, Schroeder DJ, Kallmes DF, Kadirvel R (2017). Statins are not associated with short-term improved aneurysm healing in a rabbit model of unruptured aneurysms. J Neurointerv Surg.

[153] Fahed R, Gentric JC, Salazkin I, Gevry G, Raymond J, Darsaut TE (2017). Flow diversion of bifurcation aneurysms is more effective when the jailed branch is occluded: an experimental study in a novel canine model. J Neurointerv Surg.

[154] Miyamoto T, Kung DK, Kitazato KT, Yagi K, Shimada K, Tada Y (2017). Site-specific elevation of interleukin-1β and matrix metalloproteinase-9 in the Willis circle by hemodynamic changes is associated with rupture in a novel rat cerebral aneurysm model. J Cereb Blood Flow Metab.

[155] Adibi A, Eesa M, Wong JH, Sen A, Mitha AP (2017). Combined endovascular coiling and intra-aneurysmal allogeneic mesenchymal stromal cell therapy for intracranial aneurysms in a rabbit model: a proof-of-concept study. J Neurointerv Surg.

[156] Tutino VM, Rajabzadeh-Oghaz H, Chandra AR, Gutierrez LC, Schweser F, Preda M (2018). 9.4T magnetic resonance imaging of the mouse circle of Willis enables serial characterization of flow-induced vascular remodeling by computational fluid dynamics. Curr Neurovasc Res.

[157] Greim-Kuczewski K, Berenstein A, Kis S, Hauser A, Killer-Oberpfalzer M (2018). Surgical technique for venous patch aneurysms with no neck in a rabbit model. J Neurointerv Surg.

[158] Marotta TR, Riina HA, McDougall I, Ricci DR, Killer-Oberpfalzer M (2018). Physiological remodeling of bifurcation aneurysms: preclinical results of the eCLIPs device. J Neurosurg.

[159] Li ZF, Fang XG, Zhao R, Yang PF, Huang QH, Liu JM (2018). Stromal cell-derived factor 1α facilitates aneurysm remodeling in elastase-induced rabbit saccular aneurysm. Cytokine.

[160] Fahed R, Darsaut TE, Kotowski M, Salazkin I, Raymond J (2018). Re-treatment of residual aneurysms after flow diversion: an experimental study. Neuroradiol J.

[161] King RM, Brooks OW, Langan ET, Caroff J, Clarençon F, Tamura T (2018). Communicating malapposition of flow diverters assessed with optical coherence tomography correlates with delayed aneurysm occlusion. J Neurointerv Surg.

[162] Caroff J, Tamura T, King RM, Lylyk PN, Langan ET, Brooks OW (2018). Phosphorylcholine surface modified flow diverter associated with reduced intimal hyperplasia. J Neurointerv Surg.

[163] Marosfoi M, Clarencon F, Langan ET, King RM, Brooks OW, Tamura T (2018). Acute thrombus formation on phosphorilcholine surface modified flow diverters. J Neurointerv Surg.

[164] Miyata H, Shimizu K, Koseki H, Abekura Y, Kataoka H, Miyamoto S (2019). Real-time imaging of an experimental intracranial aneurysm in rats. Neurol Med Chir (Tokyo).

[165] Liu Y, Zheng Y, An Q, Song Y, Leng B (2019). Optical coherence tomography for intracranial aneurysms: a new method for assessing the aneurysm structure. World Neurosurg.

[166] Simgen A, Tomori T, Bomberg H, Yilmaz U, Roth C, Reith W (2019). Intravenous versus intra-arterial digital subtraction angiography: occlusion rate and complication assessment of experimental aneurysms after flow diverter treatment in rabbits. Interv Neuroradiol.

[167] Strange F, Sivanrupan S, Gruter BE, Rey J, Taeschler D, Fandino J (2019). Fluorescence angiography for evaluation of aneurysm perfusion and parent artery patency in rat and rabbit aneurysm models. J Vis Exp.

[168] Ikedo T, Kataoka H, Minami M, Hayashi K, Miyata T, Nagata M (2019). Sequential inward bending of arterial bifurcations is associated with intracranial aneurysm formation. World Neurosurg.

[169] Wang GX, Xia C, Liu J, Cui C, Lei S, Gong MF (2019). The relationship of arterial wall enhancement ratio on MRI with the degree of inflammation in a rabbit aneurysm model: a pilot study. Acad Radiol.

[170] Grüter BE, Täschler D, Rey J, Strange F, Nevzati E, Fandino J (2019). Fluorescence video angiography for evaluation of dynamic perfusion status in an aneurysm preclinical experimental setting. Oper Neurosurg (Hagerstown).

[171] Nishi H, Ishii A, Ono I, Abekura Y, Ikeda H, Arai D (2019). Biodegradable flow diverter for the treatment of intracranial aneurysms: a pilot study using a rabbit aneurysm model. J Am Heart Assoc.

[172] Iseki S, Mitome-Mishima Y, Ogino I, Suga Y, Yatomi K, Nonaka S (2019). Histological and transmission electron microscopy results after embolization with hydrosoft/hydroframe coils in experimental swine aneurysm. Biomed Res Int.

[173] King RM, Marosfoi M, Caroff J, Ughi GJ, Groth DM, Gounis MJ (2019). High frequency optical coherence tomography assessment of homogenous neck coverage by intrasaccular devices predicts successful aneurysm occlusion. J Neurointerv Surg.

[174] Rajabzadeh-Oghaz H, Chandra AR, Gutierrez L, Schweser F, Ionita C, Siddiqui A, et al. (2019) High-resolution MRI of the mouse cerebral vasculature to study hemodynamic-induced vascular remodeling. SPIE Medical Imaging 10953. 10.1117/12.2511772

[175] Wanderer S, Waltenspuel C, Grüter BE, Strange F, Sivanrupan S, Remonda L (2020). Arterial pouch microsurgical bifurcation aneurysm model in the rabbit. J Vis Exp.

[176] Lyu Y, Luo J, Zhang Y, Wang C, Li A, Zhou Y (2020). An effective and simple way to establish elastase-induced middle carotid artery fusiform aneurysms in rabbits. Biomed Res Int.

[177] Fries F, Maßmann A, Tomori T, Yilmaz U, Kettner M, Simgen A (2020). Accuracy of optical coherence tomography imaging in assessing aneurysmal remnants after flow diversion. J Neurointerv Surg.

[178] Sun X, Zheng X, Zhang X, Zhang Y, Luo G (2020). Exosomal microRNA-23b-3p from bone marrow mesenchymal stem cells maintains T helper/Treg balance by downregulating the PI3k/Akt/NF-κB signaling pathway in intracranial aneurysm. Brain Res Bull.

[179] Koseki H, Miyata H, Shimo S, Ohno N, Mifune K, Shimano K (2020). Two diverse hemodynamic forces, a mechanical stretch and a high wall shear stress, determine intracranial aneurysm formation. Transl Stroke Res.

[180] Ho JP, Galex IA, Sadeghi NB, Weledji N, Cabello Bermudez SI, Mitchell BA (2021). Rabbit elastase aneurysm: imaging and histology correlates for inflammation and healing. World Neurosurg.

[181] Shimizu K, Kataoka H, Imai H, Yamamoto Y, Yamada T, Miyata H (2021). Hemodynamic force as a potential regulator of inflammation-mediated focal growth of saccular aneurysms in a rat model. J Neuropathol Exp Neurol.

[182] Vardar Z, King RM, Kraitem A, Langan ET, Peterson LM, Duncan BH (2021). High-resolution image-guided WEB aneurysm embolization by high-frequency optical coherence tomography. J Neurointerv Surg.

[183] Zhang Y, Cheng S, He Y, Tang C, Liu F, Sun Y (2021). Activated platelet-homing nanoplatform for targeting magnetic resonance imaging of aneurysm-related thrombus in rabbits. ACS Appl Mater Interfaces.

[184] Fries F, Tomori T, Schulz-Schaeffer WJ, Jones J, Yilmaz U, Kettner M (2022). Treatment of experimental aneurysms with a GPX embolic agent prototype: preliminary angiographic and histological results. J Neurointerv Surg.

[185] Hufnagl C, Broussalis E, Cognard C, Grimm J, Hecker C, Oellerer A (2022). Evaluation of a novel flow diverter, the DiVeRt system, in an animal model. J Neurointerv Surg.

[186] Wanderer S, Grüter BE, Strange F, Boillat G, Sivanrupan S, Rey J (2022). Aspirin treatment prevents inflammation in experimental bifurcation aneurysms in New Zealand White rabbits. J Neurointerv Surg.

[187] Strange F, Gruter BE, Fandino J, Marbacher S (2020) Preclinical intracranial aneurysm models: a systematic review. Brain Sci 10. 10.3390/brainsci10030134 10.3390/brainsci10030134 PMC7139747 32120907

[188] Marbacher S, Strange F, Frösén J, Fandino J (2020). Preclinical extracranial aneurysm models for the study and treatment of brain aneurysms: a systematic review. J Cereb Blood Flow Metab.

[189] Tang H, Lu Z, Xue G, Li S, Xu F, Yan Y (2020). The development and understanding of intracranial aneurysm based on rabbit model. Neuroradiology.

[190] Settecase F, Rayz VL (2021). Advanced vascular imaging techniques. Handb Clin Neurol.

[191] Murphy EA, Ross RA, Jones RG, Gandy SJ, Aristokleous N, Salsano M (2017). Imaging in vascular access. Cardiovasc Eng Technol.

[192] Thompson BG, Brown RD, Amin-Hanjani S, Broderick JP, Cockroft KM, Connolly ES (2015). Guidelines for the management of patients with unruptured intracranial aneurysms: a guideline for healthcare professionals from the American Heart Association/American Stroke Association. Stroke.

[193] Pelz DM, Fox AJ, Vinuela F (1985). Digital subtraction angiography: current clinical applications. Stroke.

[194] Howard BM, Hu R, Barrow JW, Barrow DL (2019). Comprehensive review of imaging of intracranial aneurysms and angiographically negative subarachnoid hemorrhage. Neurosurg Focus.

[195] Ruedinger KL, Schafer S, Speidel MA, Strother CM (2021). 4D-DSA: development and current neurovascular applications. AJNR Am J Neuroradiol.

[196] Rubin GD, Leipsic J, Joseph Schoepf U, Fleischmann D, Napel S (2014). CT angiography after 20 years: a transformation in cardiovascular disease characterization continues to advance. Radiology.

[197] Fleischmann D, Chin AS, Molvin L, Wang J, Hallett R (2016). Computed tomography angiography: a review and technical update. Radiol Clin North Am.

[198] White PM, Wardlaw JM, Easton V (2000). Can noninvasive imaging accurately depict intracranial aneurysms? A systematic review. Radiology.

[199] De Leucio A, De Jesus O (2022) MR angiogram. In: StatPearls Publishing, Treasure Island 32644410

[200] Kiruluta AJM, González RG (2016). Magnetic resonance angiography: physical principles and applications. Handb Clin Neurol.

[201] Zanaty M, Chalouhi N, Starke RM, Jabbour P, Hasan D (2016). Molecular imaging in neurovascular diseases: the use of ferumoxytol to assess cerebral aneurysms and arteriovenous malformations. Top Magn Reson Imaging.

[202] Ali MFA (2021). Transcranial Doppler ultrasonography (uses, limitations, and potentials): a review article. Egyptian Journal of Neurosurgery.

[203] Acuna A, Berman AG, Damen FW, Meyers BA, Adelsperger AR, Bayer KC (2018). Computational fluid dynamics of vascular disease in animal models. J Biomech Eng.

[204] Atif M, Ullah H, Hamza MY, Ikram M (2011). Catheters for optical coherence tomography. Laser Phys Lett.

[205] Aumann S, Donner S, Fischer J, Müller F (2019) Optical Coherence Tomography (OCT): Principle and technical realization. In: High Resolution Imaging in Microscopy and Ophthalmology: New Frontiers in Biomedical Optics. Springer, Cham, pp 59–85 32091846

[206] Refaat A, Yap ML, Pietersz G, Walsh APG, Zeller J, Del Rosal B (2022). In vivo fluorescence imaging: success in preclinical imaging paves the way for clinical applications. J Nanobiotechnology.

[207] Ota H, Takase K, Rikimaru H, Tsuboi M, Yamada T, Sato A (2005). Quantitative vascular measurements in arterial occlusive disease. Radiographics.

[208] Raman A, Uprety M, Calero MJ, Villanueva MRB, Joshaghani N, Villa N (2022). A systematic review comparing digital subtraction angiogram with magnetic resonance angiogram studies in demonstrating the angioarchitecture of cerebral arteriovenous malformations. Cureus.

[209] Cogswell PM, Rischall MA, Alexander AE, Dickens HJ, Lanzino G, Morris JM (2020). Intracranial vasculature 3D printing: review of techniques and manufacturing processes to inform clinical practice. 3D Print Med.

[210] Frösen J, Piippo A, Paetau A, Kangasniemi M, Niemelä M, Hernesniemi J (2004). Remodeling of saccular cerebral artery aneurysm wall is associated with rupture: histological analysis of 24 unruptured and 42 ruptured cases. Stroke.

[211] Morel S, Diagbouga MR, Dupuy N, Sutter E, Braunersreuther V, Pelli G (2018). Correlating clinical risk factors and histological features in ruptured and unruptured human intracranial aneurysms: the Swiss AneuX study. J Neuropathol Exp Neurol.

[212] Wang X, Zhu C, Leng Y, Degnan AJ, Lu J (2019). Intracranial aneurysm wall enhancement associated with aneurysm rupture: a systematic review and meta-analysis. Acad Radiol.

[213] Onwuzu SWI, Ugwu AC, Mbah GCE, Elo IS (2021). Measuring wall shear stress distribution in the carotid artery in an African population: computational fluid dynamics versus ultrasound Doppler velocimetry. Radiography (Lond).

[214] Puvanasunthararajah S, Fontanarosa D, Wille ML, Camps SM (2021). The application of metal artifact reduction methods on computed tomography scans for radiotherapy applications: a literature review. J Appl Clin Med Phys.

[215] Davis B, Royalty K, Kowarschik M, Rohkohl C, Oberstar E, Aagaard-Kienitz B (2013). 4D digital subtraction angiography: implementation and demonstration of feasibility. AJNR Am J Neuroradiol.

